# Scaling of the morphology of African cities

**DOI:** 10.1073/pnas.2214254120

**Published:** 2023-02-23

**Authors:** Rafael Prieto-Curiel, Jorge E. Patino, Brilé Anderson

**Affiliations:** ^a^Complexity Science Hub Vienna, 1080, Austria; ^b^EAFIT University, Medellín 050022, Colombia; ^c^Sahel and West Africa Club Secretariat, Organisation for Economic Co-operation and Development, Paris 75016, France

**Keywords:** urban form, sustainability, Africa, energy

## Abstract

A large proportion of Africa’s infrastructure is yet to be built. Where and how these new buildings are constructed matters since today’s decisions will last for decades. The resulting morphology of cities has lasting implications for a city’s energy needs. Estimating and projecting these needs has always been challenging in Africa due to the lack of data. Yet, given the sweeping urbanization expected in Africa over the next three decades, this obstacle must be overcome to guide cities toward a trajectory of sustainability and resilience. Based on the location and surface of nearly 200 million buildings on the continent, we estimate the interbuilding distance of almost six thousand cities. Buildings’ footprint data enable the construction of urban form indicators to compare African cities’ elongation, sprawl, and emptiness. We establish the BASE model, where the mean distance between buildings is a functional relation to the number of Buildings and their average Area, as well as the Sprawl and the Elongation of its spatial arrangement. The mean distance between structures in cities—our proxy for its energy demands related to mobility—grows faster than the square root of its population, resulting from the combined impact of a sublinear growth in the number of buildings and a sublinear increase in building size and sprawl. We estimate that when a city doubles its population, it triples its energy demand from transport.

## Significance

The emptiness, elongation, and sprawl of a city have lasting implications for cities’ future energy needs. This paper creates a publicly available set of urban form indicators and estimates intercity distances. It uses footprint data of millions of buildings in Africa as well as the boundaries of urban agglomerations, street network data, and terrain metrics to detect different extension patterns in almost six thousand cities. These methods estimate the increasingly longer commutes in urban areas and the energy needed to move millions of people. Designing compact, dense, and better-connected urban forms will help cities be more sustainable and liveable.

The world’s future is in cities. Projections estimate almost 7 out of 10 people will live in a city by 2050. Whilst many parts of the world have already undergone urbanization, the next three decades will bring sweeping changes in African cities. An additional 950 million will become urbanites by 2050 in Africa, compared to 574 million people in 2015. More people will need more buildings, future homes, schools, hospitals, markets, and other daily life stops. Where and how these buildings are constructed matters since today’s decisions will last for decades. The resulting morphology of cities—in other words, whether it is sprawling or compact, monocentric or polycentric, fragmented or contingent, densely or sparsely populated—has lasting implications for a city’s energy demand from transport (1–7).

As cities become even larger, the scaling impact of the city size on economic, social, and infrastructure indicators will become more crucial. For example, large cities tend to attract more migrants and highly skilled individuals and have higher levels of wealth and innovation but are also prone to more crime, congestion, road accidents, and some diseases (6, 8–14). Although the impact of city size in terms of some indicators remains unclear for example, in terms of CO_2_ emissions (14–17) and some indicators rely on how cities are defined, urban scaling studies provide a way to quantify the impact of city size on urban indicators (18–20). Here, we analyze the scaling impact of population size on urban form and the challenge of accommodating the growing number of people in a more efficient and sustainable way. Urban morphology goes hand-in-hand with accessibility, sustainability, car-dependency, and energy demands from transport (21, 22). So far, efforts to tackle these questions have been waylaid by the lack of data. Isolated analyses of sprawl or fragmentation using population density data of major African cities exist, but smaller and intermediary cities are often excluded. This is troubling since most of the future urbanization in the forthcoming decades will arise in these cities. Much of the collective knowledge of urban form in African cities and future energy needs is based on samples of only a few cities. However, recent advancements in data availability open new ways of observing patterns in cities at a global scale (23–26).

To address these data gaps, we capitalize on the newly available Google AI Africa Open Buildings dataset, which maps the location and area of every building in most regions of the continent (*SI Appendix*, Appendix A). We combine the infrastructure data with building’s height data modeled by the German Aerospace Centre (DLR) (27, 28), with street network metrics (29) and with terrain metrics (30). With the granularity of these datasets, combined with prior work that maps the geographical location and boundaries of nearly 6,000 urban agglomerations (31) and our models of urban form, it is possible to estimate the magnitude of African cities’ future transport energy demand.

## 1. Results

**Fig. 1.**
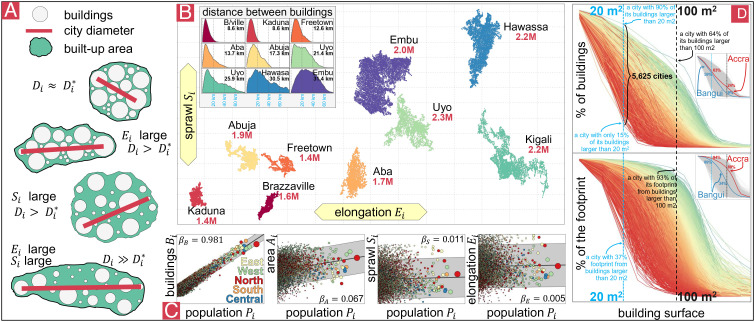
(*A*) Cities may be elongated, sprawling, or both, depending on the location of their buildings. (*B*) Footprint of buildings observed across nine cities with a similar population (between 1.4 and 2.3 million inhabitants). Cities with an elongated shape have a larger diameter meaning that the mean distance between buildings is longer. Cities with more sprawl tend to have more space between buildings, contributing to longer distances. (*C*) Observed increase in the number of buildings *B*_*i*_, their area *A*_*i*_, the sprawl *S*_*i*_, and the elongation *E*_*i*_ as the population of the city increases. (*D*) Cumulative fraction of the number of buildings (*Top*) and surface (*Bottom*) of a city formed by buildings bigger than some area. Highlighted are the observed curves in Accra and in Bangui.

Here, we construct a set of indicators to characterize urban morphology based on the geographical distribution and size of millions of buildings in African cities (32). The objective is to relate the morphology of cities to distance indicators (e.g., sprawl, elongation, and polycentricity) and the energy demand from transport (21, 33). We use the coordinates and surface of 183 million buildings constructed in nearly 6,000 cities in Africa (*SI Appendix*, Appendix A). For each city identified by Africapolis (31), we measure the mean interbuilding distance, which is our proxy for the energy demand from transport. Based on the expression of the average distance between two points inside a circle, a functional equation for the mean distance *D*_*i*_ between two buildings inside a city *i* is given by,

[1] $$\begin{array}{c} D_{i}=\frac{128}{45\pi }\sqrt{B_{i}A_{i}S_{i}E_{i}}, \end{array}$$

where *B*_*i*_ is the number of buildings and *A*_*i*_ is their average area. *S*_*i*_ is the sprawl index, which is the space between buildings, where smaller values come from a compact city. *E*_*i*_ is the elongation of a city or anisometry (7), where smaller values indicate a round shape and higher values suggest a more elongated footprint (*SI Appendix*, Appendix B). The sprawl *S*_*i*_ and elongation *E*_*i*_ are comparable across cities of different sizes, based on the maximum distance between buildings (Fig. 1).

Eq. **1** is inspired by the formula for the average distance between any two points inside a circle with area *a*, given by $128\sqrt{a}/\left(45\pi \right)$. Instead of considering only the urban footprint, the BASE model decomposes intracity distances into four multiplicative components (34, 35). The model gives an estimate for distances between buildings if a city is round and compact, *D*_*i*_^⋆^, obtained in Eq. **1** with *E*_*i*_ = *S*_*i*_ = 1, reflecting how distances increase due to the city’s footprint (number and size of buildings). We define the fragmentation of a city as $\psi_{i}\;=\;D_{i}/D_{i}^{⋆}\;=\;\sqrt{S_{i}E_{i}}\;\geq \;1$, the ratio between the observed and minimum distances, in other words, the extent to which a city departs from a packed pattern (e.g., the space between buildings: green space, blue space, roads). More fragmented cities have longer distances due to factors other than more or bigger buildings. Since there is some uncertainty when identifying the existence of buildings, we conducted a sensitivity analysis that removed buildings with low reliability and resampling and detected negligible variation in the urban form indicators. Therefore, any differentiation between the urban form indicators is not the result of that uncertainty (*SI Appendix*, Appendix C). We use cities’ elongation, sprawl, and fragmentation to detect what contributes to longer distances and characterize cities’ morphology across Africa.

The number of buildings within a city grows with population, with roughly one extra building for every 2.6 people. However, that number decreases slightly with size, reflecting a shared infrastructure. At a continental level, a city with ten times the population has 9.6 times the number of buildings (*SI Appendix*, Appendix F), so the number of buildings in a city grows sublinearly (10, 36, 37). Put together, all the buildings’ footprint comprises 10,000 constructed km^2^ (roughly the surface of Lebanon). The majority are small residential buildings (68.5% of the buildings are less than 50 m^2^, and only 0.3% have an area bigger than 500 m^2^). However, big buildings, although small in quantity, might represent a large part of the constructed surface of a city (*SI Appendix*, Appendix G). Buildings in African cities have an average surface of 55 m^2^. However, the mean area of buildings also varies with city size. Roughly 18% of the constructed surface of Africa is buildings larger than 250 m^2^. In Abidjan, for example, only 5% of the buildings are bigger than 250 m^2^, but they total 30% of the constructed surface of the city (Fig. 1*D*). Building’s average size scales with the city’s population. A city with ten times the population has buildings that are, on average, 17% bigger, the result of a disproportionate presence of big buildings in larger cities. The same applies across the whole continent. In West Africa, for instance, a city with ten times the population has buildings that are 40% bigger. Large cities in the US and OECD countries are denser (13), but this is only observed in North Africa. In the rest of African regions, the footprint of a city increases superlinearly since cities have fewer buildings per person, but those buildings tend to be bigger. In West Africa, for instance, a city with ten times the population has 12.5 times the footprint. Further, larger cities have slightly taller buildings with a higher volume per person than smaller cities, mainly because the footprint is larger (*SI Appendix*, Appendix I)

If large cities were just a scaled version of small cities, the mean distance between buildings should grow with the square root of their population (*β*_*D*_ = 0.5). However, distances in large cities grow slightly faster, with exponent *β*_*D*_ = 0.532. Also, results range between *β*_*C*_ = 0.400 for cities in Central Africa to *β*_*N*_ = 0.574 for cities in North Africa. We observe that people occupy more space at an individual level in North Africa, which contributes to the faster growth in distances between buildings, exacerbating negative land use consequences (38). Even though, cities in North Africa are likely also expanding vertically, especially as the population of a city reaches above 100,000 (39). That extra space results from fewer but larger buildings and more elongated and sprawled cities. The same is not true across the rest of the continent. In West Africa, for example, larger cities are less elongated and have a smaller sprawl. Having a smaller elongation and sprawl slightly counter the expanding number of buildings in large metropolitan areas. Cities face two opposing forces as they grow. People demand more infrastructure, so more and larger buildings. However, as the population increases, distances grow and become critical, so cities experience intense competition for space and make better use of it, which results in less elongated cities (40). The mean distance between buildings is, on average, 2.8 times larger because of the noncircular shape of African cities. However, in West, South, and Central Africa, larger cities are more compact (smaller sprawl *S*) and tend to be less elongated (smaller *E*), so even if they occupy more space, they settle in a more efficient manner (*SI Appendix*, Appendix F).

**Fig. 2.**
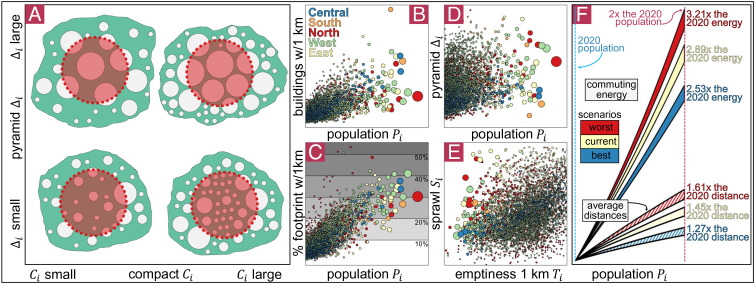
(*A*) The center of a city, identified as its most dense location, and its 1-km vicinity is formed by some buildings that may be bigger than the rest of the city (forming a pyramid city) or even smaller (forming a valley), and the vicinity might have a small or large footprint. It is possible to characterize cities by looking only at the city center. (*B*–*D*) More populous cities have more and bigger buildings by their center, so the footprint near the center is also bigger. Among cities of more than one million inhabitants, at least 30% of its center is built. (*E*) Most cities (except for some cities mostly in East Africa) have less sprawl with lower levels of emptiness. Thus, efficient use of space near the city center is crucial for reducing city sprawl.

Our proxy for a city’s energy demand from transport *T*_*i*_ is the product of its population and mean distances, so *T*_*i*_  ∝  *P*_*i*_*D*_*i*_  ∝  *P*_*i*_^1.532^ for the continent, a quantity that captures population and urban form simultaneously (21). A city with ten times the population will demand 34 times more energy for transport, and this estimate even ignores the impact of induced traffic demand (*SI Appendix*, Appendix J). Although small cities often lack urban expansion strategies, it is in large cities where the burden of sprawl and elongation is more significant. The top 1% largest cities in Africa (in terms of population), 50 cities, are home to 40% of the continent’s population, but account for 80% of the energy demand from transport, according to our estimates. Furthermore, some African countries, such as Niger or Chad, will double their 2020 population before 2050 (25). Due to the urbanization process and population growth, African cities will keep growing at an unprecedented speed. For example, Lagos might reach a population of 80 or even 100 million inhabitants (41). Assuming the population is the most important determinant for cities, we analyze the evolution of its form as cities grow (6, 24). With a population of 80 million inhabitants, and if present trends continue, a city could have 18 million buildings (roughly two-thirds of the number of buildings there are today in Nigeria) with a footprint of 175 km^2^ and an average distance between buildings above 85 km (*SI Appendix*, Appendix J). The burden on city dwellers of such an urbanization process could force the city to grow in a roughly circular, compact, and more vertical shape, urbanizing green and blue areas. As cities grow, more people will suffer from longer distances leading to higher energy demand from transport. We analyze what will happen for a city that doubles its 2020 population in terms of its footprint, distances, and energy required for transport (Fig. 2*F*). By the time African cities double their 2020 population, the average distance between buildings will increase 45%, but more people will experience longer distances, so the energy demand from transport in those cities will be three times the current levels. A city that doubles its population will experience at least 27% longer distances (up to an increase of 61% in the worst-case scenario, with high elongation and sprawl) and will require up to 3.21 times more energy demand from transport (*SI Appendix*, Appendix J). African cities will need to develop mass transport and to reduce the number of daily journeys and the spatial mismatch in order to avoid excessive congestion and increasing energy demand (42, 43).

**Fig. 3.**
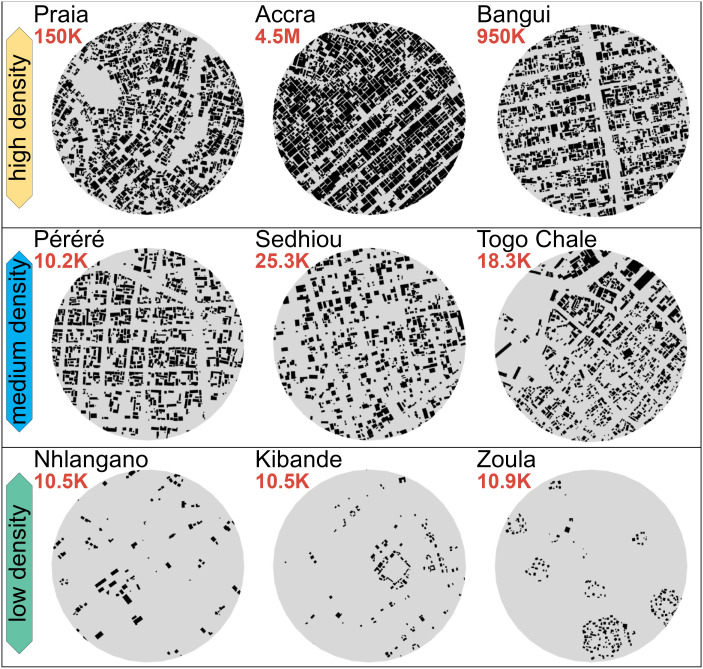
Visualization of urban footprint located within a 250-m buffer from the maximum buildings’ footprint density point for a selected group of cities. *Top*: examples of urban agglomerations with very high buildings’ footprint density at their maximum density points. *Middle*: urban agglomerations with buildings’ footprint density values close to the continental average. *Bottom*: urban agglomerations with very low buildings’ footprint density at their maximum density points. The two-dimensional kernel density was computed with a bandwidth of 250 m.

It is possible to characterize cities by observing only their densest location in terms of buildings (Fig. 2). The emptiness of the densest point increases its sprawl and the energy demand from the transport of cities (*SI Appendix*, Appendix H), which may result from a town having only a limited number of (mostly small) constructions. The densest point in a city with more than 1 million people has 30 or 40% of the surface built-up, as opposed to small towns with less than 100,000 inhabitants, where less than 10% has been constructed (Fig. 3). Thus, the sprawl at the densest point in a city with ten times more people is 63% smaller. The densest point of a city might be “empty,” meaning that its constructed surface might be small, suggesting that the whole urban area has a very low density. City size is also related to the structure of a city by its center. Buildings close to the densest point tend to have a bigger footprint in a larger city (Fig. 2). For example, a city with ten times more population has 22% bigger buildings near the center (*SI Appendix*, Appendix I). Also, most cities expand vertically for their densification process (35). Considering the total volume within the densest point across cities, we find that larger cities are more constructed and expand vertically with taller buildings. Larger cities have more footprint and are more vertical within their densest point. A city with ten times more population has 3.4 times more infrastructure volume nearby its densest location since they have more constructed surface and buildings that are 25% taller (*SI Appendix*, Appendix I).

The physical terrain is crucial to understand why cities grow into elongated or sprawled patterns, therefore, some distances between buildings will be unavoidable. Urban areas with greater altitude variation—an indicator of rugged terrain—tend to be more elongated and have greater sprawl than urban areas built on flat terrain (*SI Appendix*, Appendix D). Rugged terrain and steep slopes pose a natural barrier to building roads or taller buildings along with other infrastructure. The urban footprint adjusts to the location of usable land, resulting in a more organic and less compact urban shape. Political boundaries are also critical in terms of urban form. Cities near an international border are growing faster than other cities and tend to be more elongated (44). The street network is also not uniform across the continent. More elongated and sprawled cities have street networks with longer streets, even after controlling for population size and topography (*SI Appendix*, Appendix D). At a regional level, North African cities have the lowest average street lengths across the continent (82 m), less than half of Central African cities (211 m), making them more walkable and liveable. Cities are increasingly characterized by polycentricity, that is, the presence of multiple interconnected centers (45–47). Here, we measure polycentrism by constructing the contour tree of the density of the footprint of buildings within a city and comparing each branch’s number and volume (more details in the Methods section, *Measuring Polycentrism*). Results show that polycentric cities tend to have an elongated shape and greater sprawl and experience longer distances (*SI Appendix*, Appendix E).

## 2. Discussion

The richness of buildings’ data enables the characterization of African cities’ urban morphology at a level of granularity that has never before been possible, using the BASE model. The elongation and the sprawl of a city are indicators based on the average (geodesic) distance between buildings. We characterize urban morphology with multiplicative components, enabling us to quantify the scaling impact of city size and detect that larger cities tend to have fewer buildings per person, but they tend to have a slightly bigger footprint. Observing some attributes of a whole city might be challenging due to the lack of data, but we also show that it is possible to characterize cities’ urban morphology solely on the building size and footprint at the densest point of a city. Many cities worldwide will have roughly the same size in 2050 as their size today and, therefore, will have a similar form as well. However, this is not the case in Africa and South Asia, where cities are still experiencing their boom. Most African cities will double their 2020 population and could triple their energy demand from transport before 2050, if present trends continue. Even assuming that the share of motorized trips remains constant, that cities do not increase their automobile dependence and ignoring the additional costs of congestion and induced traffic, Africa’s energy demand from transport will increase substantially in the upcoming years but so will their time spent commuting (33, 48).

Accelerating urbanization toward a more compact, mobility-oriented, and mixed land use is associated with greater sustainability and resilience, elsewhere in the world. Concerning transport, these urban forms have shorter blocks and more connected street networks, facilitating the use of public transport and nonmotorized modes, such as walking and cycling. Transport-oriented development in hand with mixed land use and limiting new urban expansion to compact and dense developments would also help to improve sustainability by avoiding car-dependency (21, 22). However, steering urban growth toward a compact form can place pressure on green spaces, diminishing the resilience of cities to floods, heatwaves, and landslides, along with losing other ecosystem services provided by these spaces, like pollution and carbon absorption, and biodiversity conservation. The negative impacts of urbanization regarding pollution depend on how much population lives in cities and also on the area they cover, and a similar process is observed in terms of a city’s energy demand from transport (17).

Expanding and never-ending flat urban polygons are not a sustainable pathway for cities, but a sea of skyscrapers is not necessarily the answer either, since this can lead to inequality. Cities need to balance equity, cost, and sustainability—namely, materials demand from buildings, embodied carbon, as well as future energy demand. This may change across climates and regions within Africa. A one-size fits all solution is impossible and cities will likely fall on a spectrum of horizontal to vertical.

Such advancements are imperative so cities can guide urbanization toward a pathway of resilience and sustainability in the forthcoming decades. Our results show that future energy demand from transport could be incredibly cumbersome if trends continue, but also that the time spent on commuting could be a huge burden for its population. The magnitude of this burden, along with how needs are met (with or without fossil fuels), will set the future course of emissions, pollution, congestion, noise—and ultimately, the liveability of cities (49).

These data are publicly available for local actors on the OECD/SWAC’s mapping-africa-transformations.org/ and updated over time with future releases of the raw data. Freely accessible data helps local actors in African cities to track the evolution of the built environment and guide decision-making, policies, funding, and regulations in these cities, in addition to fostering peer learning and shared experiences. Climate change is a global challenge, but mitigation and adaptation rest on the shoulders of local actors.

## 3. Methods

### A. Data Construction.

**Fig. 4.**
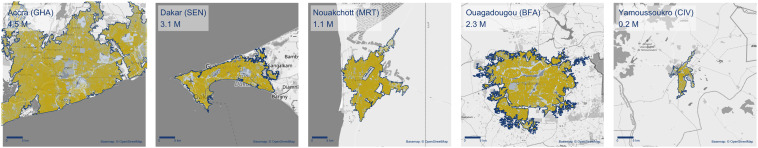
Google Open Buildings locations over Africapolis urban agglomeration boundaries. Examples from 5 capital cities in West Africa: Accra (Ghana), Dakar (Senegal), Nouakchott (Mauritania), Ouagadougou (Burkina Faso), and Yamoussoukro (Ivory Coast). Maps at the same spatial scale.

Defining urban agglomerations is challenging and often depends on considerations and parameters (18–20, 50). Africapolis applies the same definition for an urban agglomeration at a continental level (i.e., an agglomeration of at least 10,000 inhabitants and buildings less than 200 m apart), enabling it to analyze and compare cities between different countries (31). Buildings’ locations were extracted from the recently launched Google Open Buildings dataset, https://sites.research.google/open-buildings/. The buildings’ footprints were obtained using a deep learning model with high-resolution satellite imagery (50 cm pixel size) (27). We extract the buildings’ footprints located within Africapolis polygons and keep the attributes of the building’s center location (latitude and longitude), the confidence score, and the building footprint area. We assign each building the unique identifier of the Africapolis urban agglomeration to which it belongs. This way, we obtained the location and attributes of the buildings’ footprint of 6,849 African urban agglomerations (Fig. 4). We computed street network metrics (29, 51) and terrain metrics (30), such as the difference in elevation between the highest and lowest point, the average slope, and the average height within each Africapolis urban agglomeration polygon (*SI Appendix*, Appendix A).

### B. The BASE Model of Cities.

For city *i* with population size *P*_*i*_, we compute the mean distance between pairs of buildings, *D*_*i*_. Values of *D*_*i*_ are larger due to four reasons: 1) city *i* has more buildings, 2) its buildings are bigger, 3) buildings are arranged diffusely, and 4) the city has an elongated shape. Here, we capture the four factors contributing to a city having longer distances and characterize them depending on distinct city attributes, such as differences in elevation and city size. Let *B*_*i*_ be the number of buildings in the city *i* and *A*_*i*_ be their average size in m^2^. Let *S*_*i*_ >  0 be a coefficient for the diffusion of buildings, and *E*_*i*_ ≥ 1 be a coefficient that captures how elongated is the shape of the city (Fig. 1). Cities mainly grow from the bottom-up and adjust to the topography, barriers, and road infrastructure, so in general, the shape of cities is not circular (40). Our data enable us to measure the mean distance *D*_*i*_, the number of buildings *B*_*i*_, and their size *A*_*i*_. We construct *S*_*i*_ and *E*_*i*_ for each city. With values of *E*_*i*_ close to one, the city’s shape is nearly circular, and higher values represent more elongated shapes. With small values of *S*_*i*_, the city has a small sprawl, so buildings are arranged compactly. Thus, with small *E*_*i*_ and *S*_*i*_, the city has roughly a circular shape and compacted buildings. The impact of *E*_*i*_ and *S*_*i*_ is to increase distances, taking a tight circle as the basis. We construct our BASE model based on the formula for the expected distance between two points inside a circle. Eq. **1** is inspired by the average distance between points inside a circle, where instead of the radius, we use the city’s footprint, *B*_*i*_*A*_*i*_, and two shape parameters with a multiplicative impact, *S*_*i*_*E*_*i*_.

Inspired by an ellipse, we measure the elongation of a city. Thus, with *E*_*i*_, we capture how “elliptical” the city’s shape is. The mean distance between two points inside an ellipse has no closed solution (52), but an approximation can be constructed by considering the ratio between the major and the minor axis. Thus, we define the elongation *E*_*i*_ as,

[2] $$\begin{array}{c} E_{i}=\frac{\sqrt{\pi }M_{i}}{2\sqrt{B_{i}A_{i}}}, \end{array}$$

where *M*_*i*_ is the “diameter” of the city, that is, the longest distance between any two buildings, and $2\sqrt{B_{i}A_{i}/\pi }$ is the smallest possible diameter of a circle with *B*_*i*_*A*_*i*_ as a footprint. Smaller values of *E*_*i*_ mean that the ratio between the smallest and the largest radius are similar, so the city’s shape is more circular. Larger values mean more elongated shapes. In Eq. **2**, *M*_*i*_ is the “major axis”, and $2\sqrt{B_{i}A_{i}/\pi }$ is the “minor axis” of a city (*SI Appendix*, Appendix B). By considering *E*_*i*_ to be the ratio between two distances, we obtain a scale-free coefficient *E*_*i*_ ≥ 1 concerning the number or area of buildings. That means that if a city is a scaled version of another, for example, with four times the number of buildings (or buildings four times the size), then distances also grow, including doubling the maximum distance and doubling the smallest diameter, so *E*_*i*_ remains the same.

We define the sprawl of a city as everything else that increases distances in cities besides the number of buildings, their area, and elongation. From Eq. **1**, we get that,

[3] $$\begin{array}{c} S_{i}=\frac{45^{2}\pi^{3/2}D_{i}^{2}}{2^{13}M_{i}\sqrt{B_{i}A_{i}}}=\gamma \frac{D_{i}^{2}}{M_{i}\sqrt{B_{i}A_{i}}}, \end{array}$$

for *γ* = 45^2^*π*^3/2^/2^13^ ≈ 1.38.

Imagine that instead of buildings of a city, we have toy bricks of different sizes. There are infinitely many configurations to arrange those bricks keeping the maximum distance fixed (with a fixed diameter), meaning many brick configurations have the same elongation. In one extreme, the bricks “fill” the area with the corresponding major axis, but in the other extreme, the largest distance is observed only between a few bricks. Therefore, the sprawl *S*_*i*_ captures those possible brick arrangements.

Our technique decomposes the mean distance between buildings into four multiplicative components. Two components (*B*_*i*_ and *A*_*i*_) are measured directly from the data, and we have constructed a mathematical expression for *S*_*i*_ and *E*_*i*_, the sprawl and elongation. Thus, we have an exact expression that equates the mean distance between buildings in a city with four urban indicators, Eq. **4**.

[4] $$D_{i}=\frac{128}{45\pi }{\left(\underset{\text{footprint}}{\underset{︸}{B_{i}A_{i}}}\underset{\text{shape}}{\underset{︸}{S_{i}E_{i}}}\right)}^{1/2}.$$

One way to interpret the elongation and the sprawl of a city is that the mean distance between buildings in cities increases proportionally to $\sqrt{E_{i}}$, so if a city has an elongation value of *E*_*i*_ = 4, then the mean distance between buildings is double because the city is elongated, and similarly for the sprawl *S*_*i*_.

### C. Minimum Distances and Fragmentation.

The number of buildings in a city is the most obvious reason why distances grow in large cities. More people means more buildings which then translates into larger distances. The same occurs with building size, so we “discount” those two reasons. Eq. **1** enables us to detect how small distances could be if the city was compact and had a circular shape, with area *B*_*i*_*A*_*i*_ and with *E*_*i*_ = 1 and *S*_*i*_ = 1. The smallest mean distance *D*_*i*_^⋆^ is given by,

[5] $$\begin{array}{c} D_{i}^{⋆}=\frac{128}{45\pi }\sqrt{B_{i}A_{i}}. \end{array}$$

We construct the fragmentation index *ψ*_*i*_ of a city, by

[6] $$\begin{array}{c} \psi_{i}=\frac{D_{i}}{D_{i}^{⋆}}=\sqrt{S_{i}E_{i}}, \end{array}$$

that indicates how distances in city *i* are longer due to an elongated shape and more spacing between buildings. Fragmentation is a coefficient *ψ*_*i*_ ≥ 1 independent of the number and area of buildings.

### D. Measuring Polycentrism.

Measuring polycentrism is based on three methodological stages: delineating urban regions, identifying subcenters, and then applying some mathematical function to obtain an index for a city (45). Different techniques have been used based on infrastructure data, such as satellite images, the density of points of interest, road network, or buildings data (53–56) and also based on mobility data or travel surveys (46, 57, 58). Measuring polycentrism depends not only on the type of data and the criteria applied for defining centers and subcenters but also on the possibly divergent methods of measuring polycentricity (47).

The kernel density estimates the intensity of the number of buildings and the constructed surface per unit area (59). For example, the technique has been used for constructing a continuous surface representing the intensity of human activity based on points of interest in a city (55). The kernel density is a cumulative function obtained by adding a decaying surface for each building. Formally, for some point *x* in space, we define the kernel as,

[7] $$\begin{array}{c} k\left(x\right)=\sum_{j=1}^{n}w_{j}f\left(d_{x,j}\right), \end{array}$$

where *n* is the number of buildings, *w*_*j*_ are the weights of each building, taken to be its area, and *d*_*x*, *j*_ is the distance between the point *x* and the centroid of the building, and *f* is a decreasing function, known as the smoothing kernel, here taken to be a Gaussian function. The result is a surface over each urban area that highlights parts with more or larger constructions (peaks) considered centers of the city (60).

Based on the kernel density, a contour adjacent relation tree is constructed (61), where each node on the tree is a new contour. Contour trees represent spatial relationships between the contours of the kernel surface and summarize relationships at different levels (62). Separate peaks (urban centers) are identified as branches on a tree, which are connected depending on the different contour levels of the surface. The procedure gives *N* branches, where *N* = 1 is a monocentric city. Each branch has three indicators: height (corresponding to the kernel estimate), area (representing the total surface of the city that belongs to that branch), and volume (obtained by multiplying the area and height of each branch).

Let *Br*_*i*_ be the number of branches of city *i* and let *v*_*k*_ be the volume of each branch in decreasing order (so that *v*_1_ ≥ *v*_2_ ≥ … ≥ *v*_*Br*_*i*__). The polycentrism index *ϕ*_*i*_ is defined as,

[8] $$\begin{array}{c} \phi_{i}=\frac{1}{v_{1}}\sum_{k=1}^{Br_{i}}kv_{k}. \end{array}$$

**Fig. 5.**
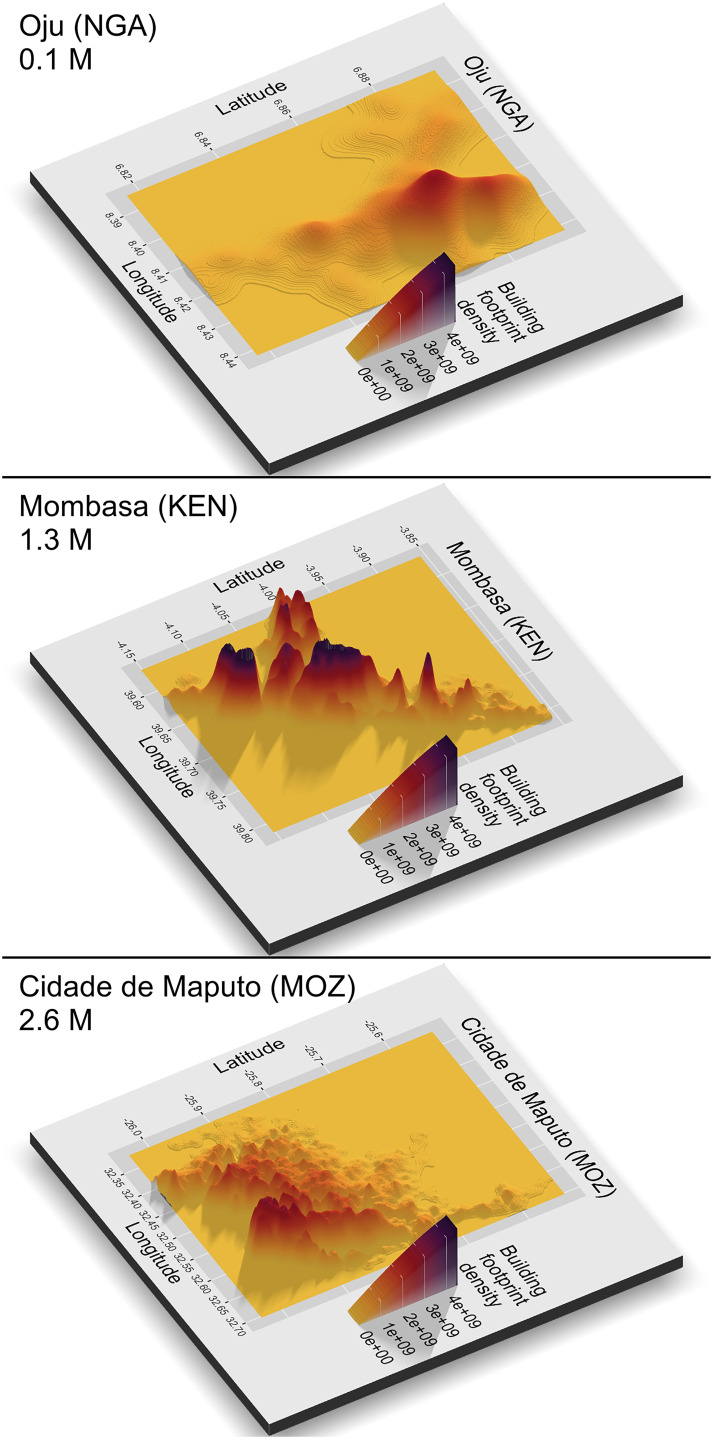
Three-dimensional representation of buildings’ footprint density. *Top*: Oju (Nigeria), a monocentric city with low levels of elongation and sprawl; *Middle*: Mombasa (Kenya), a polycentric city with high levels of elongation but low levels of sprawl; *Bottom*: Maputo (Mozambique), a polycentric city with a low level of elongation but a high level of sprawl. Each city is plotted at a different spatial scale 3D plots created using rayshader package (63) in R.

The index *k* inside the sum of Eq. **8** helps increase the value of *ϕ*_*i*_ with the number of branches. By dividing by *v*_1_ in Eq. **8**, a comparable index across cities is obtained (*SI Appendix*, Appendix E). If *ϕ*_*i*_ = 1 then *i* is monocentric. A city with two distant and equal-sized centers (so that they belong to different branches) has *ϕ*_*i*_ = 3 (with three equal-sized centers *ψ*_*i*_ = 6, and so on). The procedure also gives the volume tree of a city, where the dimension of each branch (horizontal axis) and its height (vertical axis) represent the centers of the city (61, 64). Larger cities tend to be more polycentric, formed by more branches with more separation between them and a larger relative volume. However, medium-sized cities may also be highly polycentric, reflecting fragmented urban areas (Fig. 5).

### E. Impact of City Size.

The infrastructure and the socioeconomic outputs of a city vary according to many factors, and city size has been detected to be a critical aspect of cities (6, 10, 36). We characterize the four city indicators of the BASE model and detect if they vary according to city size. We fit equations like *B*_*i*_ = *αP*_*i*_^*β*^ between the number of buildings *B*_*i*_ and the population *P*_*i*_ for some coefficients *α* and *β*, and similarly for the other indicators. Values of *β* ≈ 0 indicate that city size has little or no impact on the corresponding indicator. Values of *β* ≈ 1 indicate a linear growth, and values below (and above) *β* = 1 are a sublinear (or superlinear) impact of city size (*SI Appendix*, Appendix F).

The scaling coefficients show a sublinear relation with the number of buildings *B*_*i*_ (so fewer buildings per person as cities grow), sublinear with the area of buildings *A*_*i*_ (so bigger buildings in larger cities). Also, larger cities tend to have slightly more sprawl, and the elongation is statistically the same across cities of different sizes (Table 1).

**Table 1.** Observed scaling coefficients at the continental level

Variable	*α*		*β*	
*B*_*i*_ - number of buildings	0.423	0.029	0.981	(0.007)
*A*_*i*_ - area of buildings	24.85	1.29	0.067	(0.005)
*S*_*i*_ - sprawl	0.725	0.067	0.011	(0.006)
*E*_*i*_ - elongation	5.925	0.337	0.005	(0.005)
*D*_*i*_ - mean distance	6.126	0.479	0.532	(0.006)

Many historical and geographical differences across regions and countries in Africa also impact how cities have grown. Thus, the impact of city size is not uniform across the continent. For example, in West Africa, buildings are bigger in larger cities (so, a city with ten times the population has buildings that are 40% bigger). In contrast, in South Africa, buildings tend to be the same size across all cities (Table 2). Cities in Central and South Africa become rounder and more compact as they grow, as opposed to cities in North Africa, where elongation and sprawl increase in larger cities.

**Table 2.** Observed scaling coefficients across regions in Africa

	North	West	East	Central	South
*B*_*i*_	0.926^* * *^	0.957^* * *^	1.042^* * *^	0.990^* * *^	1.005^* * *^
	(0.013)	(0.011)	(0.010)	(0.017)	(0.014)
*A*_*i*_	0.044^* * *^	0.144^* * *^	0.035^* * *^	0.076^* * *^	0.018
	(0.009)	(0.010)	(0.007)	(0.012)	(0.010)
*S*_*i*_	0.106^* * *^	−0.026^**^	0.026^*^	−0.156^* * *^	−0.078^* * *^
	(0.012)	(0.009)	(0.013)	(0.014)	(0.019)
*E*_*i*_	0.071^* * *^	−0.017^*^	−0.001	−0.109^* * *^	−0.065^* * *^
	(0.010)	(0.007)	(0.010)	(0.015)	(0.019)
*D*_*i*_	0.574^* * *^	0.528^* * *^	0.551^* * *^	0.400^* * *^	0.440^* * *^
	(0.012)	(0.008)	(0.011)	(0.012)	(0.022)
*ψ*_*i*_	0.089^* * *^	−0.022^**^	0.013	−0.133^* * *^	−0.072^* * *^
	(0.010)	(0.008)	(0.011)	(0.013)	(0.018)
*ϕ*_*i*_	0.310^* * *^	0.186^* * *^	0.325^* * *^	0.103^* * *^	0.259^* * *^
	(0.009)	(0.006)	(0.010)	(0.008)	(0.014)
*A*100_*i*_	0.078^* * *^	0.201^* * *^	0.122^* * *^	0.204^* * *^	0.048^**^
	(0.014)	(0.013)	(0.013)	(0.024)	(0.017)
*θ*_*i*_	0.970^* * *^	1.100^* * *^	1.078^* * *^	1.066^* * *^	1.023^* * *^
	(0.014)	(0.009)	(0.011)	(0.018)	(0.018)
*θ*1*km*_*i*_	0.408^* * *^	0.471^* * *^	0.413^* * *^	0.515^* * *^	0.402^* * *^
	(0.013)	(0.010)	(0.012)	(0.019)	(0.022)

^***^P < 0.001; ^**^P < 0.01; ^*^P < 0.05.

The mean distance between buildings is also affected by city size. Using Eq. **1** to compute the scaling coefficient between buildings is also possible. Since $D_{i}=\frac{128}{45\pi }\sqrt{B_{i}A_{i}S_{i}E_{i}}$, then we can write *D*_*i*_ as it varies with city size and obtain that *D*_*i*_ = *γP*_*i*_^(*β*_*B*_ + *β*_*A*_ + *β*_*S*_ + *β*_*E*_)/2^, with $\gamma =\frac{128}{45\pi }\sqrt{\alpha_{B}\alpha_{A}\alpha_{S}\alpha_{E}}$. Thus, the scaling coefficient of distances is obtained by half the sum of the scaling of buildings, area, sprawl, and elongation (*SI Appendix*, Appendix J). For example, in North Africa, the scaling coefficient of the distance is _*N*_*β*_*D*_ = (0.926 + 0.044 + 0.106 + 0.071)/2 = 0.574 so the mean distance between buildings in cities increases at a faster rate than the square root of the population. Having decomposed the mean distance into four urban components enables us to detect that in North Africa, the reasons why distances are longer in larger cities are first the number of buildings, then the increasing sprawl and elongation, and to a small extent, having bigger buildings. But in South Africa, having more and bigger buildings in cities increases distances in larger cities, but a decreasing sprawl and elongation contribute to reducing distances. In South Africa, the mean distance between buildings in cities increases at a slower rate than the square root of the population, with _*S*_*β*_*D*_ = 0.440. Suppose larger cities were just a scaled version of a small city. In that case, the number of buildings per person should be constant (so *β*_*B*_ = 1), and the rest of the scaling coefficients should be zero, reflecting the same building size, sprawl, and elongation, so that the mean distance should grow with the square root of the population. However, a large city is not just a scaled version of a small city. Most components and aspects of a city vary with its size.

Beyond distances, it is possible to decompose a city’s footprint and fragmentation. The footprint *θ*_*i*_ can be decomposed as *θ*_*i*_ = *B*_*i*_*A*_*i*_ = *α*_*θ*_*P*^*β*_*B*_ + *β*_*A*_^, so for example, the footprint is superlinear in West Africa but sublinear in North Africa. The fragmentation *ψ*_*i*_ can be decomposed as $\sqrt{S_{i}E_{i}}=\alpha_{\psi }P_{i}^{(\beta_{S}+\beta_{E})/2}$, where $\alpha_{\psi }=\sqrt{\alpha_{S}\alpha_{E}}$ and where the population has a scaling coefficient (*β*_*S*_ + *β*_*E*_)/2. Thus, larger cities in North Africa are more fragmented than smaller cities mainly due to a higher sprawl, but the opposite happens in Central Africa, where the sprawl and elongation are smaller in larger cities, so they tend to be less fragmented (*SI Appendix*, Appendix F).

### F. Characterizing Cities Based on the City Center.

Considering the distribution of the urban footprint at a distance *R* = 1 km from its center enables us to observe the sprawl across cities based on the same shape. Comparing the same shape across different urban polygons means we can ignore the elongation and focus only on the sprawl. The sprawl of a city inside a circle, referred to as the emptiness *T*_*i*_^(*R*)^ ≥ 1, is defined as the ratio between the surface of the city center and the part that is constructed, given by,

[9] $$\begin{array}{c} T_{i}^{\left(R\right)}=\frac{\pi R^{2}}{B_{i}^{\left(R\right)}A_{i}^{\left(R\right)}}, \end{array}$$

where *B*_*i*_^(*R*)^ and *A*_*i*_^(*R*)^ are the number and average area of buildings inside that circle. The emptiness *T*_*i*_^(*R*)^ corresponds to the observed sprawl inside a circle, and a higher emptiness means that a smaller surface of the center has been constructed (*SI Appendix*, Appendix H). Although longer distances than *R* = 1 km may also be considered for observing cities at their center, we observe that a circle with a 1 km radius is the largest circle that fits almost always inside the polygon of all cities. Bigger circles often are not fully contained inside the polygon of cities, so analyzing the footprint depends on some elongation, whereas smaller circles have a smaller building sample in cities.

## Supplementary Material

Appendix 01 (PDF)

## Data Availability

Data can be downloaded through the Mapping Africa Transformations Platform (MAPTA) website: https://mapping-africa-transformations.org/ and the code and data can also be downloaded at https://github.com/rafaelprietocuriel/UrbanFragmentation.
