# Supplementary material for: Scaling of the morphology of African cities
Source: Proc Natl Acad Sci U S A. 2023 Feb 23;120(9):e2214254120. doi: 10.1073/pnas.2214254120 (PMC9992857; doi:10.1073/pnas.2214254120)
Supplement: Supplementary file 1 — Appendix 01 (PDF) [file pnas.2214254120.sapp.pdf]

SCALING OF THE MORPHOLOGY OF AFRICAN CITIES

Rafael Prieto-Curiel
Complexity Science Hub Vienna
Josefstaedter Strasse 39
1080 Vienna, Austria
prieto-curiel@csh.ac.at

Jorge E. Patino
Research in Spatial Economics
Universidad EAFIT
Medellín, Antioquia, Colombia

Brilé Anderson
Sahel and West Africa Club - OECD
2 Rue André Pascal
75016 Paris, France

1 Appendix

1.a Data construction

We use open geospatial data sets for this analysis: urban boundaries, buildings' locations, street network data, and elevation data. The boundaries of African urban agglomerations were downloaded from the Africapolis website in November 2021 [1]. The downloaded dataset contains 7720 features with polygon geometry representing urban agglomeration boundaries by 2015, packed in ESRI Shapefile format. Defining metropolitan areas is challenging and often depends on certain considerations and parameters. Africapolis applies the same definition for a metropolitan area, enabling us to compare cities at a continental level. According to the Africapolis website, an urban agglomeration unit combines satellite and aerial imagery and official demographic data such as censuses and other cartographic sources. The delineation of an urban agglomeration boundary is based on a spatial approach that combines physical limits, the continuity of the built-up area, and demographic criteria to provide a standardised continental spatial definition of urban areas. All urban agglomerations considered have more than 10,000 inhabitants.

Buildings' locations were extracted from the recently launched Google Open Buildings dataset, `https://sites.research.google/open-buildings/` which contains 516M building footprints across an area of 19.4M km^2, 64% of the African continent [2]. The buildings' footprints were obtained using a deep learning model with high-resolution satellite imagery (50 cm pixel size) [2]. This dataset is packed into 136 different files in comma-separated values format. It contains information on the location of the building's footprint centre in geographic coordinates (latitude and longitude), its area in m^2, a *Plus Code* corresponding to the centre of the building, and a confidence score that informs about the confidence level of the building detection, and the geometry of the building's footprint. See `https://maps.google.com/pluscodes/` for more information on Plus Codes.

We downloaded the 136 Open Buildings files (49.8 GB) and implemented a geoprocessing workflow in R software [3] to extract the buildings' footprints within Africapolis polygons. We keep the attributes of the building's centre location (latitude and longitude), the confidence score, and the building footprint area. We assign the unique identifier (agglosID) of the Africapolis urban agglomeration it belongs. This way, we obtained the location and attributes of the buildings' footprints of 6,849 African urban agglomerations. There are 871 Africapolis urban agglomerations without Google Open Building data. In Morocco, Western Sahara, Libya, Chad, Cameroon, and South Sudan, as well as North Kivu Province (Democratic Republic of Congo), Cabo Delgado Province (Mozambique), and the Southwestern region in Sudan, all lack buildings' footprints in the Google Open Buildings dataset. We also combine the buildings' footprint with data provided by the German Aerospace Centre, giving the average height and volume of different regions of the city [4].

We computed street network metrics (the average street length and the total number of nodes or intersections) using the Python library OSMnx [5] with the Africapolis urban agglomeration polygons. We used that information to compute the number of buildings per street node for each urban agglomeration. We also calculated some terrain metrics, such as the difference in elevation between the highest and lowest point, the average slope, and the average height within each Africapolis urban agglomeration polygon using the NASADEM digital elevation model in Google Earth Engine [6].

Since our procedure combines different geographical data, other filters are applied to the cities:

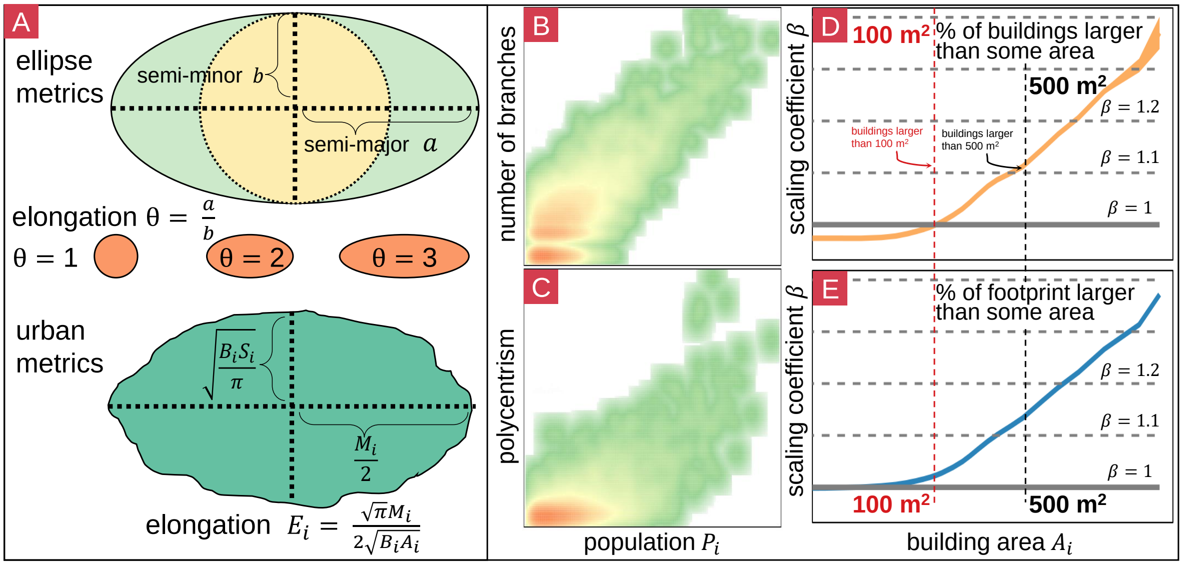

Figure 1: A - The (modified) eccentricity of an ellipse e' is defined as the ratio between the semi-major and semi-minor axis, both squared. Similarly, we define the city eccentricity θ_i as the ratio between the maximum observed distance (taken as the major axis) and the minimum radius of a circle with B_i buildings with an average surface A_i. B, C - Number of branches (top) and polycentrism (bottom) observed in a city according to its population (horizontal axis). D, E - Scaling coefficient β (vertical) of the number of buildings larger than some area (top) for different areas (horizontal axis). Scaling coefficient β (vertical) of the area in the city constructed based on buildings larger than some area (top) for different areas (horizontal axis).

○ Drop 1,202 cities with less than 2,000 buildings since there is not enough data in the urban area.

○ Drop 123 cities with elongation $E_i > 20$ and seven cities with sprawl $S_i > 4$, reflecting that polygons and buildings do not spatially align. The mean elongation is $\mu_E = 7.5$ with $\sigma_E = 4.5$, and the mean sprawl is $\mu_S = 0.94$ with $\sigma_S = 0.83$, so cities with a higher elongation or sprawl are outliers.

○ Drop 24 cities with a footprint per person above 60 m^2. The mean footprint per person is $\mu_\theta = 19$ m^2 per person, with $\sigma_\theta = 20$.

1.b Measuring the elongation and sprawl of a city

Based on the eccentricity of an ellipse, we construct a metric using the ratio between the "major" and "minor" axis of a city. The major axis of a city is M_i, the maximum observed distance between buildings. If all city buildings were arranged next to each other in a circular shape, they would form a circle with area $B_i A_i$ and with radius $\sqrt{B_i A_i / \pi}$, considered to be the minor axis of a city. There is no closed formula to measure the mean distance between two points inside an ellipse [7]. A good approximation is given by $128b\theta/(45\pi)$, where $\theta = \sqrt{a/b}$, and where a is the major axis a and b is the minor axis of the ellipse, so θ captures the discrepancy between a and b. The formula is similar to the distance between two points inside a circle of radius b but multiplied by a factor $\theta \geq 1$ that captures the shape of the ellipse. The formula is exact for $\theta = 1$ and works well for small values of θ. Therefore, in an ellipse, distances grow roughly proportional to θ. For city i, we construct its elongation E_i as the ratio between the maximum observed distance between buildings and the minimum possible city radius. As with an ellipse, values of E_i close to one mean a more circular shape, whereas higher values mean elongated cities across some axis. Also, distances in the city are expected to grow proportional to $\sqrt{E_i}$, so the expression aligns with the $BASE$ model (Figure 1-A).

Results show a scaling impact of city size that increases the number of buildings, their area, and the city's sprawl and elongation (Table 1).

Further, we detect that some urban indicators beyond population also alter the urban footprint and shape, including the number of buildings (Table 2), area (Table 3), sprawl (Table 4) and elongation (Table 5) of cities.

	$y = \log B$	$y = \log A$	$y = \log S$	$y = \log E$	$y = \log D$	$y = \log \theta$
(Intercept)	-0.846^{***}	-10.603^{***}	-0.322^{***}	1.779^{***}	-5.095^{***}	2.366^{***}
	(0.068)	(0.052)	(0.063)	(0.054)	(0.061)	(0.067)
log(Population_2015)	0.981^{***}	0.067^{***}	0.011	0.005	0.532^{***}	1.048^{***}
	(0.007)	(0.005)	(0.006)	(0.005)	(0.006)	(0.006)
R^2	0.799	0.031	0.001	0.000	0.594	0.825
Adj. R^2	0.799	0.031	0.000	−0.000	0.594	0.825
Num. obs.	5625	5625	5625	5625	5625	5625

$^{***}p < 0.001$; $^{**}p < 0.01$; $^{*}p < 0.05$

Table 1: Scaling of the $BASE$ components, distance D and of footprint θ.

	$y = \log B$
(Intercept)	$2.4331\ (0.0979)^{***}$
log(Population_2015)	$0.5675\ (0.0089)^{***}$
RegionEast	$0.0821\ (0.0182)^{***}$
RegionNorth	$0.0655\ (0.0211)^{**}$
RegionSouth	$0.5718\ (0.0230)^{***}$
RegionWest	$0.1285\ (0.0192)^{***}$
log(Polycentrism)	$0.4115\ (0.0150)^{***}$
log(dif_elev_m)	$0.1140\ (0.0057)^{***}$
a100	$0.1232\ (0.0586)^{*}$
TotalFootprintCentre1km	$0.0000\ (0.0000)^{***}$
MeanArea1000m	$-0.0144\ (0.0005)^{***}$
log(street_length_avg)	$-0.0433\ (0.0093)^{***}$
IsPyramid Δ	$0.6257\ (0.0231)^{***}$
R^2	0.9175
Adj. R^2	0.9173
Num. obs.	5625

$^{***}p < 0.001$; $^{**}p < 0.01$; $^{*}p < 0.05$

Table 2: Model number of buildings B depending on the population, region, polycentrism, differences in elevation, the area covered by buildings with more than 100 m^2 (a100), the footprint within the centre and the area of buildings, the average street length and the ratio between the average area of buildings within 1 km of the city centre and the average area of buildings in the city (IsPyramid Δ).

	$y = \log A$
(Intercept)	$-10.372\ (0.026)^{***}$
log(Population_2015)	$-0.008\ (0.002)^{***}$
RegionEast	$0.001\ (0.005)$
RegionNorth	$-0.058\ (0.006)^{***}$
RegionSouth	$-0.032\ (0.006)^{***}$
RegionWest	$-0.071\ (0.005)^{***}$
log(Polycentrism)	$-0.004\ (0.004)$
log(dif_elev_m)	$0.008\ (0.002)^{***}$
a100	$0.877\ (0.016)^{***}$
TotalFootprintCentre1km	$0.000\ (0.000)^{***}$
MeanArea1000m	$0.010\ (0.000)^{***}$
log(street_length_avg)	$0.009\ (0.004)^{*}$
log(BuildingsPerNode)	$-0.005\ (0.002)^{**}$
IsPyramid Δ	$-0.370\ (0.006)^{***}$
R^2	0.951
Adj. R^2	0.951
Num. obs.	5625

$^{***}p < 0.001$; $^{**}p < 0.01$; $^{*}p < 0.05$

Table 3: Model building area A depending of the population, region, polycentrism, differences in elevation, the area covered by buildings with more than 100 m^2 (a100), the footprint within the centre and the area of buildings, the average street length and the ratio between the average area of buildings within 1 km of the city centre and the average area of buildings in the city (IsPyramid Δ).

	$y = \log S$
(Intercept)	-2.510 $(0.099)^{***}$
log(Population_2015)	0.025 $(0.010)^{*}$
RegionEast	0.013 (0.017)
RegionNorth	0.064 $(0.020)^{**}$
RegionSouth	0.091 $(0.022)^{***}$
RegionWest	-0.034 (0.018)
MeanBuildingArea	1521.843 (1073.992)
log(Polycentrism)	0.399 $(0.015)^{***}$
log(dif_elev_m)	0.049 $(0.006)^{***}$
a100	0.039 (0.063)
TotalFootprintCentre1km	-0.000 $(0.000)^{***}$
MeanArea1000m	-0.002 $(0.001)^{**}$
log(street_length_avg)	0.295 $(0.013)^{***}$
log(nodes)	0.114 $(0.006)^{***}$
IsPyramid Δ	0.223 $(0.033)^{***}$
ICoast	0.071 $(0.014)^{***}$
IBorder	0.058 $(0.017)^{***}$
R^2	0.578
Adj. R^2	0.576
Num. obs.	5625

$^{***}p < 0.001$; $^{**}p < 0.01$; $^{*}p < 0.05$

Table 4: Model sprawl S depending of the population, region, the mean building area, polycentrism, differences in elevation, the area covered by buildings with more than 100 m^2 (a100), the footprint within the centre and the area of buildings, the average street length and the ratio between the average area of buildings within 1 km of the city centre and the average area of buildings in the city (IsPyramid Δ), whether the city is coastal and whether the city is within an international border (less than 50 km).

	$y = \log E$
(Intercept)	-0.211 $(0.088)^{*}$
log(Population_2015)	0.022 $(0.008)^{**}$
RegionEast	0.064 $(0.015)^{***}$
RegionNorth	-0.072 $(0.018)^{***}$
RegionSouth	-0.189 $(0.020)^{***}$
RegionWest	-0.109 $(0.016)^{***}$
MeanBuildingArea	907.518 (949.954)
log(Polycentrism)	0.218 $(0.013)^{***}$
log(dif_elev_m)	0.078 $(0.005)^{***}$
a100	0.071 (0.056)
TotalFootprintCentre1km	-0.000 $(0.000)^{***}$
MeanArea1000m	-0.002 $(0.001)^{*}$
log(street_length_avg)	0.275 $(0.012)^{***}$
log(nodes)	0.092 $(0.005)^{***}$
IsPyramid Δ	0.085 $(0.029)^{**}$
ICoast	0.050 $(0.012)^{***}$
IBorder	0.037 $(0.015)^{*}$
R^2	0.548
Adj. R^2	0.546
Num. obs.	5625

$^{***}p < 0.001$; $^{**}p < 0.01$; $^{*}p < 0.05$

Table 5: Model elongation E depending on the population, region, the mean building area, polycentrism, differences in elevation, the area covered by buildings with more than 100 m^2 (a100), the footprint within the centre and the area of buildings, the average street length and the ratio between the average area of buildings within 1 km of the city centre and the average area of buildings in the city (IsPyramid Δ), whether the city is coastal and whether the city is within an international border (less than 50 km).

1.c Model uncertainty

There are two sources of uncertainty related to the urban form metrics. The first is the uncertainty related to the data used for the analysis and the process of detecting buildings in a city. And the second source is related to the metrics designed to quantify urban forms and their potential sensitivity to extreme values. Regarding how buildings are identified, the Google Open Buildings dataset contains a confidence score indicating the certainty that each building exists and a traffic light rating label based on that score. It is constructed by averaging the confidence gained for each pixel that forms the base of the building. It depends on many elements, including the quality of the images and the contrast between buildings and their surrounding. The data only contains buildings with a confidence higher than 0.6. Buildings with confidence between 0.60 and 0.65 are labelled as "red", between 0.65 and 0.70 are labelled as "yellow" and above 0.70 are labelled "green". For all cities, we estimate the mean distance between all buildings and the mean distance considering only constructions with at least a medium confidence (so yellow and green-labelled buildings) and constructions with high confidence (only green-labelled buildings). We do the same for the maximum distance between buildings. The motivation behind our procedure is to detect whether a city's mean or maximum distance is based mostly on reliable data. Results (Figure 2-A) show that in 99% of the cities, the mean distance

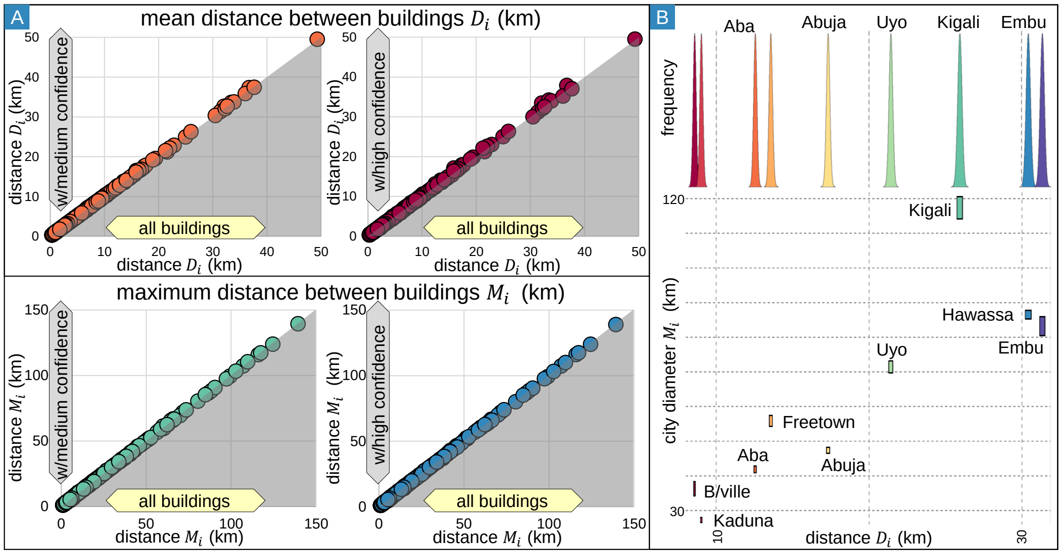

Figure 2: A - Observed distance between buildings (top) and maximum distance (bottom) when all buildings are considered (in the horizontal axis) against some filtering procedure (in the vertical axis) depending on the level of certainty of each building. B - Observed distribution of the average distance (top) and the average and maximum distance (bottom) when 30,000 pairs of buildings are randomly sampled for the set of cities. The sampling procedure was repeated 1,000 times, and, for each city, the top and bottom 2.5% observations were dropped to remove possible outliers.

between buildings varies less than 4% when buildings with at least medium confidence are considered and varies less than 8% when only buildings with high confidence are considered. Similarly, for 99% of the cities, the maximum distance between buildings varies less than 3% when buildings with at least medium confidence are considered and varies less than 4% when only buildings with high confidence are considered. Only in three cities in Egypt with a population of fewer than 20,000 inhabitants, the average distance has a slightly wider variation. Still, in one of those cities, for instance, the average distance between buildings is 467 m, with a maximum distance of 1804 m when all buildings are considered, and an average distance of 488 m with a maximum distance of 1726 m when buildings with medium and high confidence are considered (so a variation of 4.5% on the mean distance and of 4.3% on the maximum distance). Therefore, distance metrics have a small variation when only some of the buildings are considered. This is mainly since the confidence in the buildings does not have a strong spatial bias within a single urban polygon. Thus, considering only buildings with some confidence score acts mostly as a sampling process.

The second source of uncertainty relates to the metrics and their sensitivity to extreme values of the average or maximum distance between buildings. For example, detecting only a few buildings constructed along some road could substantially increase the maximum distance within a city. We test this scenario by taking nine cities with a population between 1.4 and 2.3 million people and then randomly taking 30,000 pairs of buildings inside each city. We measure their mean and their maximum sampled distance. We repeat this procedure 1,000 times by sampling different pairs of buildings each time (Figure 2-B). The motivation here is that if the metrics in any city depend only on a few buildings, results will vary drastically between different samplings. Results show that the mean distance in Kigali, one of the selected cities, varies from 25,973 m to 26,034 m across the many samples. Although there is some uncertainty in the mean and maximum distance observed in cities and the elongation and the sprawl, their variation is small. In all cases and for all nine cities, the metrics vary less than 2.5% from their original values and are far from overlapping. Thus, Kigali, for example, has very long mean distances and maximum distance between buildings, and that number is statistically distinct from the one observed in Uyo, even if both cities are of similar size.

1.d Terrain, elevation and physical factors

The growth of cities is often bounded by physical barriers, such as mountains or coasts. Thus, when we observe that a city is very elongated, it is frequently because of rugged terrain and differences in elevation or because it is located beside a water body (river, lake, or sea). To measure the impact of physical barriers and rough terrain on the elongation of a city, we measure the distance to the nearest coast, the point with the lowest and the highest altitude in the city, and differences in altitude. This set of variables enables us to correlate the city metrics and detect whether being close to

	$y = \log \psi$
(Intercept)	$-1.360\ (0.081)^{***}$
log(Population_2015)	$0.023\ (0.008)^{**}$
RegionEast	$0.039\ (0.014)^{**}$
RegionNorth	$-0.004\ (0.017)$
RegionSouth	$-0.049\ (0.018)^{**}$
RegionWest	$-0.072\ (0.015)^{***}$
MeanBuildingArea	$1214.681\ (878.774)$
log(dif_elev_m)	$0.063\ (0.005)^{***}$
a100	$0.055\ (0.052)$
TotalFootprintCentre1km	$-0.000\ (0.000)^{***}$
MeanArea1000m	$-0.002\ (0.001)^{**}$
log(Polycentrism)	$0.308\ (0.012)^{***}$
log(street_length_avg)	$0.285\ (0.011)^{***}$
log(nodes)	$0.103\ (0.005)^{***}$
IsPyramid Δ	$0.154\ (0.027)^{***}$
ICoast	$0.061\ (0.011)^{***}$
IBorder	$0.048\ (0.014)^{***}$
R^2	0.622
Adj. R^2	0.620
Num. obs.	5625

$^{***}p < 0.001$; $^{**}p < 0.01$; $^{*}p < 0.05$

Table 6: Model fragmentation ψ depending of the population, region, the mean building area, polycentrism, differences in elevation, the area covered by buildings with more than 100 m^2 (a100), the footprint within the centre and the area of buildings, the average street length and the ratio between the average area of buildings within 1 km of the city centre and the average area of buildings in the city (IsPyramid Δ), whether the city is coastal and whether the city is within an international border (less than 50 km).

	$y = \log \phi$
(Intercept)	$-4.004\ (0.074)^{***}$
log(Population_2015)	$0.319\ (0.008)^{***}$
RegionEast	$0.163\ (0.016)^{***}$
RegionNorth	$0.330\ (0.018)^{***}$
RegionSouth	$0.195\ (0.020)^{***}$
RegionWest	$0.204\ (0.017)^{***}$
MeanBuildingArea	$-8458.903\ (981.017)^{***}$
TotalFootprintCentre1km	$-0.000\ (0.000)^{***}$
MeanArea1000m	$0.009\ (0.001)^{***}$
log(dif_elev_m)	$0.027\ (0.005)^{***}$
a100	$-0.098\ (0.058)$
log(street_length_avg)	$0.151\ (0.012)^{***}$
log(nodes)	$0.061\ (0.006)^{***}$
IsPyramid Δ	$-0.223\ (0.030)^{***}$
ICoast	$0.062\ (0.013)^{***}$
IBorder	$-0.004\ (0.016)$
R^2	0.529
Adj. R^2	0.528
Num. obs.	5625

$^{***}p < 0.001$; $^{**}p < 0.01$; $^{*}p < 0.05$

Table 7: Model polycentrism ϕ depending of the population, region, the mean building area, differences in elevation, the area covered by buildings with more than 100 m^2 (a100), the footprint within the centre and the area of buildings, the average street length and the ratio between the average area of buildings within 1 km of the city centre and the average area of buildings in the city (IsPyramid Δ), whether the city is coastal and whether the city is within an international border (less than 50 km).

a coast increases the urban form indicators. Cities with high elevation or differences in altitude suggest proximity to mountains and the presence of physical barriers and rugged terrain.

Results show that larger differences in elevation and being close to a coastline are associated with higher levels of elongation (Table 5) and sprawl (Table 4), and therefore higher levels of fragmentation (Table 6) and of polycentrism (Table 7). Our models show that an increase of 1% in elevation difference is associated with increases of 5% in sprawl and almost 8% in elongation. Being along a coastline is associated with 7% more sprawl and 5% more elongation. However, small cities along main roads also feature very high levels of elongation, even when the differences in elevation are not large. The most elongated cities in Africa are always located along a coastline or main roads.

1.e Polycentrism in cities

Cities frequently develop and grow in a polycentric manner, that is, by merging different urban areas or adding new centres. Here, we measure polycentrism using the spatial distribution of buildings, the corresponding kernel density surface and the contour tree it forms. Results show that the number of branches of the corresponding contour tree increase with city size (Figure 1-B, C). However, those new branches add a small relative volume for small cities. In

general, only cities above 100 thousand inhabitants are polycentric, where those extra branches add volume separate to the city's centre.

There is a wide variation in the levels of polycentrism in cities. Take, for example, an urban area with roughly one million inhabitants. The levels of polycentrism vary from 1 to values above 20, suggesting that it is not only size that affects the polycentric growth of cities.

Sprawling cities are more polycentric. Even though greater sprawl signals larger distances between buildings, this does not necessarily translate into longer distances travelled by a city dweller in a polycentric city. Polycentric urban forms mean that multiple city centres exist so that people can meet their needs closer to home (e.g., the "15-minute city") rather than travelling to one centre (as in a monocentric city).

1.f Impact of city size on urban form

People from larger cities tend to produce more patents, have a higher income per capita, and suffer more crime and some types of diseases [8]. People from larger cities migrate less and are more likely to return after moving [9]. Smaller cities usually require more infrastructure per person, such as the road surface or the petrol stations [8]. A mathematical expression to capture the impact of city size is obtained by adjusting the equation

$$Y_i = \alpha P_i^{\beta}, \tag{1}$$

where Y_i is the variable of interest for city i. Then, Y_i could be the number of restaurants in the city i, for example. We obtain the coefficients α and β through a regression. With $\hat{\beta} > 1$ the results are called "superlinear" and indicate that large cities have higher values of the variable Y per capita (since the per capita rate Y_i/P_i is given by $\hat{\alpha} P_i^{\hat{\beta}-1}$). With $\hat{\beta} < 1$ results are "sublinear" and with $\hat{\beta} \approx 1$ city size has little or no impact on the per capita rate of that city. Therefore, the coefficient of interest is usually β and values above or below $\beta = 1$ are critical. It has been observed that for some social indicators, $\beta = 1.15$ is frequently obtained, which means that when comparing two cities, i and j, where the population of j is ten times the population of i, then, the expected values of Y_j are approximately $10^{1.15} = 14.13$ times larger, and, on a per capita basis, city j has 1.413 more of Y than city i. Thus, comparing a city with 100,000 inhabitants and a city ten times larger, with one million people, it has 14.13 times more income, so a person from a large city has 1.413 times the income of a person from a smaller city, for example. Also, values of $\hat{\beta} \in (0, 1)$ indicate that the corresponding variable Y increases with city size but at a slower rate than population (so the per capita ratio decreases with size). Finally, the same mechanism can be used to detect if some city indicator decreases with a larger population when $\hat{\beta} < 0$.

We consider scaling with respect to city size, so we fit the equations

$$\begin{aligned}
\text{Buildings:} \quad & B_i = \alpha_B P_i^{\beta_B} \\
\text{Area of buildings:} \quad & A_i = \alpha_A P_i^{\beta_A} \\
\text{Sprawl:} \quad & S_i = \alpha_S P_i^{\beta_S} \\
\text{Elongation:} \quad & E_i = \alpha_E P_i^{\beta_E} \\
\text{Distance:} \quad & D_i = \alpha_D P_i^{\beta_D}
\end{aligned}$$

where α_B and β_B are the scaling parameters which indicate whether city size impacts the number of buildings. Values of $\beta_B > 1$ indicate that the number of buildings in a city grows faster than its population. In other words, there are more per capita buildings in larger cities. And similarly, for the other coefficients, values above one would suggest larger buildings, more urban sprawl or elongated shapes. We fit similar coefficients also for the fragmentation, the observed average distance between buildings, the polycentrism, the footprint and pyramid.

Imagine a city with n buildings, each occupied by one person, with the same area (say, 1 m^2). If buildings are arranged one next to the other to minimise the average distance between them, they would be arranged roughly circular and compactly. For a sufficiently large number of buildings, they will form approximately a circle with an area of n. The mean distance between those buildings would be $128/(45\pi)n^{1/2}$. Therefore, in a city with a circular and compact shape, distances should grow with the square root of the population. In other words, if we fit the scaling coefficient $D_i = \alpha P_i^{\beta}$, a lower boundary of β is given by $\beta \geq 1/2$. The BASE model is based on setting $D_i = \frac{128}{45\pi}\sqrt{B_i A_i S_i E_i}$, where we obtained the coefficient $\beta_D = 0.543$, so distances in cities grow at a faster rate than the boundary $(1/2)$. The city metrics vary substantially across the five African regions.

Variable	Scaling	cut-off	observed
Buildings	$B_i = \alpha_B P_i^{\beta_B}$	$\beta_B = 1$	0.980997
Area	$A_i = \alpha_A P_i^{\beta_A}$	$\beta_A = 0$	0.067196
Sprawl	$S_i = \alpha_S P_i^{\beta_S}$	$\beta_S = 0$	0.010522
Elongation	$E_i = \alpha_E P_i^{\beta_E}$	$\beta_E = 0$	0.005021
Fragmentation	$\psi_i = \alpha_\psi P_i^{\beta_\psi}$	$\beta_\psi = 0$	0.007772
Distance	$D_i = \alpha_D P_i^{\beta_D}$	$\beta_D = 1/2$	0.531868
Distance*	$D_i^\star = \alpha_{D^\star} P_i^{\beta_{D^\star}}$	$\beta_{D^\star} = 1/2$	0.52410
Polycentrism	$\phi_i = \alpha_\phi P_i^{\beta_\phi}$	$\beta_\phi = 0$	0.248818
Footprint	$\theta_i = \alpha_\theta P_i^{\beta_\theta}$	$\beta_\theta = 1$	1.048
Volume	$V_i = \alpha_V P_i^{\beta_V}$	$\beta_V = 1$	1.0238
Pyramid	$\Delta_i = \alpha_\Delta P_i^{\beta_\Delta}$	$\beta_\Delta = 0$	0.024897

	$y = \log H$
(Intercept)	$1.6623\ (0.0284)^{***}$
log(Population_2015)	$0.0204\ (0.0021)^{***}$
RegionEast	$-0.0986\ (0.0080)^{***}$
RegionNorth	$0.0917\ (0.0080)^{***}$
RegionSouth	$-0.1279\ (0.0098)^{***}$
RegionWest	$-0.0683\ (0.0077)^{***}$
log(Sprawl)	$-0.0227\ (0.0064)^{***}$
log(Elongation)	$-0.0186\ (0.0079)^{*}$
dif_elev_m	$-0.0001\ (0.0000)^{***}$
ICoast	$0.0505\ (0.0064)^{***}$
IBorder	$-0.0274\ (0.0082)^{***}$
R^2	0.3032
Adj. R^2	0.3020
Num. obs.	5583

$^{***}p < 0.001$; $^{**}p < 0.01$; $^{*}p < 0.05$

Table 8: Average building size H_i depending of the population, region, sprawl, elongation, differences in elevation, whether the city is coastal and whether the city is within an international border (less than 50 km).

1.g The size of buildings across Africa

Most buildings across Africa have a tiny footprint. A small footprint corresponds to a low-rise construction. We only have some uncertainty on the height in buildings with a large footprint. Therefore, the distribution of the footprint of buildings is also an indicator of infrastructure, including construction materials, overcrowding, and resilient structures, among many economic indicators. Most buildings, particularly in small cities, are quite small (Table 8). For example, most buildings in Bangui are smaller than 25 m^2 (something like a square with 5 m on each side). We compare the distribution of building size between cities (Table 9).

However, outliers in terms of building size are relevant, and they do alter the city indicators. For example, consider two cities with 25,000 buildings (roughly 100,000 inhabitants) each, with a similar abundance of small buildings.

	$y = a100$
(Intercept)	$-0.1562\ (0.0209)^{***}$
log(Population_2015)	$-0.0027\ (0.0020)$
RegionEast	$0.0472\ (0.0036)^{***}$
RegionNorth	$0.1099\ (0.0041)^{***}$
RegionSouth	$0.0935\ (0.0045)^{***}$
RegionWest	$0.0780\ (0.0037)^{***}$
MeanBuildingArea	$7678.7422\ (201.9923)^{***}$
log(dif_elev_m)	$-0.0036\ (0.0012)^{**}$
log(Polycentrism)	$-0.0052\ (0.0031)$
TotalFootprintCentre1km	$0.0000\ (0.0000)$
MeanArea1000m	$0.0007\ (0.0002)^{***}$
log(nodes)	$0.0131\ (0.0013)^{***}$
log(street_length_avg)	$0.0071\ (0.0028)^{*}$
IsPyramid Δ	$0.0010\ (0.0070)$
ICoast	$0.0130\ (0.0029)^{***}$
IBorder	$0.0091\ (0.0037)^{*}$
R^2	0.8979
Adj. R^2	0.8976
Num. obs.	5625

$^{***}p < 0.001$; $^{**}p < 0.01$; $^{*}p < 0.05$

Table 9: Area from buildings larger than 100 m depending on the population, region, the mean building area, differences in elevation, the footprint within the centre and the area of buildings, the average street length, the ratio between the average area of buildings within 1 km of the city centre and the average area of buildings in the city (IsPyramid Δ), whether the city is coastal and whether the city is within an international border (less than 50 km).

Observing that one of those two cities has 250 large buildings (1% of them) changes how we look at that city since it could correspond to a state capital, for instance. Also, results vary if by "large" buildings we mean with a footprint of more than 100 m^2 or more than 500 m^2. Therefore, we consider the percentage of the built-up area constructed from buildings larger than some threshold $\kappa > 0$. We vary the values of κ from 20 m^2 (tiny buildings) to 5000 m^2. To detect the impact of city size on the number of large and small buildings, we consider a threshold area ρ and measure, for city i, the number of buildings smaller than ρ, say $s_i(\rho)$, and the number of buildings larger than ρ, say $l_i(\rho)$. Then, we fit the Poisson regression

$$s_i(\rho) = \alpha_s(\rho) P_i^{\beta_s(\rho)}, \text{ and } l_i(\rho) = \alpha_l(\rho) P_i^{\beta_l(\rho)}. \tag{2}$$

For example, values of $\beta_s(\rho)$ below 1 indicate an abundance of buildings smaller than ρ in smaller cities, whereas values above 1 indicate an abundance of small buildings in larger cities. And the same of $\beta_l(\rho)$ for buildings larger than ρ. Different values of ρ result in a different division of small or large buildings. Results show that the number of buildings in a city is sublinear (so a city with 100,000 inhabitants usually has more buildings per person than a city with one million people, ten times larger), but the relationship changes with building size (Figure 1-D, E). Considering buildings with more than 100 m^2, we obtain that the scaling coefficient is approximately one, meaning the same number of buildings per person in small and large cities. As buildings grow, they become scarce in small cities, so the scaling coefficient increases. For buildings larger than 500 m^2, the scaling coefficient is $\beta_{n500} = 1.12$, meaning that for a city that is ten times the size, the number of buildings larger than 500 m^2 is $10^{1.12} = 13.2$ times larger. Thus, larger cities have bigger buildings.

The footprint of a city constructed from buildings larger than a specific size is superlinear, and that relationship increases with the threshold size (Figure 1-E). The area of a city constructed of buildings larger than 500 m^2, for example, shows a scaling coefficient of $\beta_{a500} = 1.15$, so again, if a city is ten times larger, then it has $10^{1.15} = 14.1$ times more surface of large buildings.

1.h The uneven distribution of large buildings across a city

The presence of large buildings is related to city size, but the location of those large buildings within a city also plays a role. We distinguish some cities where the "centre" has a high frequency of large buildings, and the area decays as the distance to the city centre increases, thus, forming a "pyramid" type of city. In contrast, a different kind of city is observed when large buildings are spread across the urban polygon, likely signalling factories, logistics and storage rooms. The city centre is identified as the point with the highest weighted density of buildings, obtained by a kernel density estimate of the point process formed by the coordinates of buildings, weighted by their footprint. Thus, the density surface gives the constructed area within some radius at each x, y coordinate. We then identify the city centre as the location with the highest built density. Although the city centre could be defined differently (for example, by analysing the polygon's centroid, looking for some central business district or looking at the building closer to others), our technique identifies the location in the city with the highest level of constructed surface and it is dependent only on buildings and not on the shape of the polygon.

We construct a metric that captures whether a city has a pyramid or a more flat dispersion of buildings. But we also want to consider the relative size of buildings. That is, a city might be regarded as more pyramid-like if the largest buildings in the city are by its centre, even if those buildings are relatively small. For measuring a relative factor of how the city has a pyramidal distribution, we construct an index based on the relative size of buildings in cities. The coefficient is constructed by the ratio between the average size of buildings within the city centre and the average size of buildings in that city. Formally, let μ_i be the average size of buildings in city i, and let ν_i be the average size of buildings within a radius of 1 km within the city centre. Then, the flatness coefficient Δ_i is given by

$$\Delta_i = \frac{\nu_i}{\mu_i}, \tag{3}$$

where $\Delta_i \approx 1$ means that buildings in a city have a similar footprint in the city centre. Larger values of Δ_i suggest a pyramid-type of the city, and smaller values of Δ_i suggest that the city centre has smaller buildings than the outskirts.

By comparing the average size of buildings within the densest point to the rest of the city, we obtain an index that identifies the dispersion of large buildings in a town. If the recognised centre has smaller buildings, it corresponds to a city centre with small but very compact buildings. If buildings within the city centre are of equal size to the rest of the city, it means a slight variability in the size of buildings, corresponding to a flat city. We consider the average size of buildings within a radius of 1 km within the city centre. The flatness coefficient Δ_i, combined with other aspects such as city size, mean building size and emptiness, distinguishes the internal structure of cities (Table 10).

We vary the ratio within the distance of the densest point of the city. Within a reasonable distance between 500 m and 3 km, we obtain similar results (Table 11 shows the results when considering a radius of 1 and 3 km).

	$y = \log \Delta$
(Intercept)	-0.6059 $(0.0392)^{***}$
log(Population_2015)	0.0763 $(0.0039)^{***}$
RegionEast	0.0364 $(0.0097)^{***}$
RegionNorth	-0.0301 $(0.0097)^{**}$
RegionSouth	0.0649 $(0.0128)^{***}$
RegionWest	-0.0816 $(0.0097)^{***}$
MeanBuildingArea	-1780.1001 $(131.6482)^{***}$
TotalFootprintCentre1km	-0.0000 $(0.0000)^{***}$
FootprintPerPerson	0.0032 $(0.0003)^{***}$
R^2	0.2331
Adj. R^2	0.2320
Num. obs.	5625

$^{***}p < 0.001$; $^{**}p < 0.01$; $^{*}p < 0.05$

Table 10: Ratio building size inside the city centre, Δ_i depending on the population, region, the mean building area, the footprint within the centre and the footprint per person.

	$y = \log \theta_{1km}$	$y = \log \theta_{3km}$
(Intercept)	6.4191^{***}	3.5406^{***}
	(0.0864)	(0.0859)
log(Population_2015)	0.5560^{***}	0.8415^{***}
	(0.0074)	(0.0074)
RegionEast	0.2033^{***}	0.1593^{***}
	(0.0229)	(0.0228)
RegionNorth	0.2968^{***}	0.1778^{***}
	(0.0259)	(0.0258)
RegionSouth	0.2934^{***}	0.5418^{***}
	(0.0289)	(0.0287)
RegionWest	0.2516^{***}	0.1469^{***}
	(0.0241)	(0.0239)
MeanBuildingArea	-7007.4782^{***}	-5026.2724^{***}
	(1415.9295)	(1406.6931)
log(Polycentrism)	-0.6074^{***}	-0.6226^{***}
	(0.0178)	(0.0177)
log(dif_elev_m)	0.0486^{***}	0.1111^{***}
	(0.0071)	(0.0070)
a100	0.6553^{***}	1.2729^{***}
	(0.0826)	(0.0821)
MeanArea1000m	0.0068^{***}	0.0013
	(0.0012)	(0.0012)
Capital	-0.0937	-0.3469^{***}
	(0.0635)	(0.0631)
IsPyramid Δ	-0.3640^{***}	-0.1067^{*}
	(0.0432)	(0.0429)
ICoast	0.0135	0.0729^{***}
	(0.0184)	(0.0183)
R^2	0.6233	0.8130
Adj. R^2	0.6224	0.8126
Num. obs.	5509	5509

$^{***}p < 0.001$; $^{**}p < 0.01$; $^{*}p < 0.05$

Table 11: Ratio building size inside city centre comparing a distance of $R = 1$ and $R = 3$ km. The dependent variable depends on population, region, men building area, polycentrism, differences in elevation, the area covered by buildings with more than 100 m^2 (a100), the area of buildings within the centre, whether the city is a country capital, the ratio between building size nearby the centre and in the rest of the urban polygon and whether the city is coastal.

Observing an urban area only at its densest point gives a powerful indication of what the rest of the city looks like and its urban form. The sprawl of a city inside a circle, referred to as the emptiness $T_i^{(R)} \geq 1$, is defined as the ratio between the surface of the city centre and the constructed part. A city with a high emptiness $T_i^{(R)}$ will be highly sprawled within the rest of the city (Table 12), but it will also be more elongated and, in turn, more fragmented. If the emptiness of a city is double, then the overall sprawl increases by 55%, the elongation increases by 35%, and therefore, the average distance between buildings across the whole city increases by 45%.

1.i Vertical growth and densification of cities

At the building level, perhaps due to their exposure to the sun, lower temperatures or stronger winds, carbon emissions increase with height [10]. At the neighbourhood level, areas with tall buildings use more gas, even controlling for surface area, volume or number of residents [11]. However, some of the additional costs of tall buildings are limited or compensated by other gains. For example, elevators consume roughly 1% of the annual electrical energy in New York City [12] and also, there is a smaller demand for heating with increasing density [13].

Buildings with a few floors (perhaps where even a lift is not needed) are a better compromise between prominent skyscrapers and flat but expanding urban polygons. Improving urban form does not rely on tallness alone [11]. We

	$y = \log S$	$y = \log E$	$y = \log \psi$
(Intercept)	-4.831^{***}	-1.185^{***}	-3.008^{***}
	(0.086)	(0.077)	(0.073)
log(Population_2015)	0.282^{***}	0.188^{***}	0.235^{***}
	(0.006)	(0.005)	(0.005)
$\log(T^{(R)}$	0.633^{***}	0.436^{***}	0.535^{***}
	(0.010)	(0.009)	(0.008)
RegionEast	0.156^{***}	0.163^{***}	0.159^{***}
	(0.019)	(0.017)	(0.016)
RegionNorth	0.057^{**}	-0.163^{***}	-0.053^{***}
	(0.018)	(0.017)	(0.016)
RegionSouth	0.343^{***}	-0.034	0.155^{***}
	(0.022)	(0.020)	(0.019)
RegionWest	0.005	-0.110^{***}	-0.052^{***}
	(0.018)	(0.016)	(0.015)
R^2	0.482	0.430	0.503
Adj. R^2	0.482	0.430	0.503
Num. obs.	5625	5625	5625

$^{***}p < 0.001$; $^{**}p < 0.01$; $^{*}p < 0.05$

Table 12: Impact of the emptiness of the densest point of a city on its sprawl, elongation and fragmentation.

	$y = \log V^{(R)}$
(Intercept)	$8.0045\ (0.1206)^{***}$
log(Population_2015)	$0.5521\ (0.0088)^{***}$
RegionEast	$-0.1181\ (0.0338)^{***}$
RegionNorth	$-0.4718\ (0.0341)^{***}$
RegionSouth	$0.0651\ (0.0416)$
RegionWest	$-0.1366\ (0.0328)^{***}$
log(Sprawl)	$-0.5051\ (0.0270)^{***}$
log(Elongation)	$-0.1494\ (0.0336)^{***}$
dif_elev_m	$-0.0004\ (0.0001)^{***}$
ICoast	$-0.0326\ (0.0271)$
IBorder	$0.1279\ (0.0349)^{***}$
R^2	0.5163
Adj. R^2	0.5154
Num. obs.	5583

$^{***}p < 0.001$; $^{**}p < 0.01$; $^{*}p < 0.05$

Table 13: Volume within densest location $V^{(R)}$ depending on the population, region, sprawl, elongation, differences in elevation, whether the city is coastal or nearby an international border.

analyse the vertical growth in cities in two manners. First on the aggregate surface of the city and second within 1 km of its densest point. We decompose the constructed volume of a city, V_i as the product of its footprint θ_i and its average height H_i, so $V_i = \theta_i H_i$. We write the volume, footprint and average height as a function of the population of the city, and obtain $\alpha_V P_i^{\beta_V} \propto P_i^{\beta_\theta} P_i^{\beta_H}$, so that $\beta_V = \beta_\theta + \beta_H$. We obtain that $\beta_\theta = 1.0502 \pm 0.0065$ and $\beta_H = 0.0181 \pm 0.0022$. Therefore, larger cities have more surface per person and a slightly higher building height. Combined, both effects give $\beta_V = 1.06836 \pm 0.0087$, meaning that larger cities have a slightly higher volume per person than smaller cities, but this is mostly because the footprint is larger.

Looking only at the urban footprint at a distance $R = 1$ km from its densest point, we can also decompose the volume as $V_i^{(R)} = \theta_i^{(R)} H_i^{(R)}$. We express the footprint, height and volume in terms of the population and obtain the corresponding coefficients, with $\beta_{V^{(R)}} = \beta_{\theta^{(R)}} + \beta_{H^{(R)}}$. We obtain that $\beta_{\theta^{(R)}} = 0.43766 \pm 0.0063$ and that $\beta_{H^{(R)}} = 0.0955 \pm 0.0155$. Therefore, a larger city has more footprint within the densest point and becomes more vertical. A city with ten times more population has buildings that are $10^{0.0955} = 1.25$, so 25% taller nearby the densest point of the city. Combining both effects of taller buildings and more constructed surfaces within the densest point, we obtain that $\beta_{V^{(R)}} = 0.5332$, meaning within the densest point, the constructed infrastructure increases considerably in large cities. A city with ten times more population has $10^{0.5332} = 3.413$ times more volume within the centre. However, most of the additional infrastructure is due to cities' horizontal growth, not taller buildings. Thus, even cities with a high density can still gain from vertical growth [14].

It is also possible to observe the vertical growth of cities by their highest point I_i within the urban footprint. By writing the scaling equation $I_i = \alpha_I P_i^{\beta_I}$, we get that $\beta_I = 0.2375 \pm 0.0046$, suggesting that within a large city, there are usually taller buildings. Here we obtain that the tallest building of a city is 73% taller within a city with ten times more population.

Finally, the availability of taller buildings and infrastructure is affected by specific urban indicators (Table 13). More elongated and fragmented cities and cities near an international border tend to have shorter buildings and fewer infrastructure volumes.

1.j Distance and energy demand from transport

Infrastructure and urban form are strongly linked and alter the energy demand from transport [15]. The mean distance between buildings in a city is our proxy for its energy demand from transport. Given the average distance between buildings on city i, expressed as a function of the population by $D_i(P_i) = \alpha_D P_i^{\beta_D}$ we estimate that the total energy demand from transport in the city is proportional to $T_i = \alpha_T P_i^{\beta_T} = \alpha_T P_i^{\beta_D+1}$ for some $\alpha_T > 0$. This way, P_i experiences a commuting distance in the city that is proportional to $P_i^{\beta_D}$. Having decomposed (as in [16] for the emissions of a city and as in [17] for its density) the mean distances in cities (Equation 1 in the manuscript) we get that the distances grow with the population of a city, with exponent $\beta_D = (\beta_B + \beta_A + \beta_S + \beta_E)/2$ and the energy demand from transport grows with the population with exponent $\beta_T = (\beta_B + \beta_A + \beta_S + \beta_E)/2 + 1$. Although we cannot estimate values of α_T based on our data, we observe that at a continental level, $\beta_D = 0.532$. Therefore, we estimate that the energy demand from transport in African cities grows superlinearly, with a coefficient of 1.532. Thus, a city with ten times the population requires 34 times more energy demand from transport because the city is larger, so with more and bigger buildings, distances grow.

The BASE model enables us to predict the number of buildings and the size of a city with some population P_d and estimates a city's sprawl and elongation index. Most countries in Africa will double their 2020 population, some even before 2050, and some cities might reach a population of 80 or even 100 million inhabitants [18]. Assuming that population is the only determinant for cities, we analyse the expected evolution of distances and energy demand from transport as their population increases [19, 20]. Due to the continent's demographic expansion and urbanisation process, most cities will double their population within decades. And when a city doubles in size, the total energy demand from transport in the continent will increase three times. Our estimate varies by region. In North Africa, a city that doubles its population will require 2.97 times more energy from transport, but a city in Central Africa will only require 2.63 times more energy from transport since cities have less sprawl and become less elongated as they increase in size. The opposite happens in North Africa, where larger cities tend to have higher levels of fragmentation.

Here, we are looking at a simplified system where the "cost" of commutes is measured by the average geodesic distance between two buildings inside the city. We could adjust a couple of elements to model the results more accurately. First, consider the existing street network instead of geodesic distances between buildings. However, measuring distances between pairs of cities and comparing them against road distances in Africa, it was detected that road distances could be roughly approximated by a factor of 1.38 [21]. Therefore, we could multiply the geodesic distance between each pair of buildings and obtain an approximation of the road distance. Second, we could also consider different speeds per the mode of transport and consider commuting time instead of simply distances. It is frequently argued that travel time is the strongest predictor of mode choice, so we could consider commuting time instead of road distance [22]. When a city doubles its population, there will be three elements that will increase the commuting cost: an increase in the distances (as observed by the BASE model), an increase in the people experiencing the longer distances (obtained by multiplying distances by population), and finally, more congestion. Larger cities have more road traffic, so that people will experience even more delays.

There is also an issue with the spatial arrangement of residential and activity areas (work, leisure, education, etc). In theory, even if a metropolitan area is large, people could still live close to their destinations, for example, with the 15-minute city that aims to provide all services within a 15-minute journey of all houses. However, most cities experience some spatial misalignment between residential and activity areas and those distances are frequently experienced by the low-income population. It has been argued that people switch between transport modes depending on their commuting costs or distances so that if cycling is faster than driving, the city will eventually have more cyclists, but if public transport becomes faster, it will have more users, and so on [22]. Instead of the modal share, there might also be some feedback between commuting costs, population distribution, and how the city grows. Maybe this feedback is one of the driving forces that push cities into being more round and compact by increasing the demand for more central spaces.

Further, we construct three different scenarios for cities that double their population. For the current scenario, we use the obtained values at a continental level of the coefficients β_B, β_A, β_S, and β_E, and we also consider the best-case scenario, where the values considered are the smallest observed across the five regions, consisting of a sublinear growth in the number of buildings, as observed in North Africa (with ${}_N\beta_B = 0.926$), with roughly the same building size, as observed in South Africa (with ${}_S\beta_A = 0.018$), with less sprawled and elongated cities, as observed in Central Africa (with ${}_C\beta_S = -0.156$ and ${}_C\beta_E = -0.109$). And similarly, we construct the worst-case scenario, where larger cities have more and larger buildings, but they also become more sprawled and elongated. The reasoning is that at least one of the five regions follows the best and worst scenarios. Results show that the coefficient for the best-possible scenario gives $\beta_D^b = 0.3395$, with $\beta_T^b = 1.3395$, and the coefficient for the worst-possible scenario gives $\beta_D^w = 0.6815$, with $\beta_T^w = 1.6815$. Thus, in the best-case scenario, when population doubles, distances increase $2^{0.3395} = 1.265$ times,

and the energy demand from transport increase by $2^{1.3395} = 2.53$ times. In the worst-case scenario, distances will increase by $2^{0.6815} = 1.606$ times and the energy demand from transport will increase by $2^{1.6815} = 3.206$ times.

1.k Terms summary

Elements considered in the model (Table 14).

$P_i \geq 10,000$	population in city i	count of people
$B_i \geq 2,000$	number of buildings	count of buildings
$A_i \geq 10$	average size of buildings	area in m^2
$S_i \geq 0.25$	factor for how measuring the sprawl of a city	coefficient
$E_i \geq 1$	factor for how elongated is a city	coefficient greater than 1
$D_i \geq 300$	average distance between buildings	distance in km
$D_i^\star \geq 300$	average distance if the city had perfect packing	ideal distance in km.
$\psi_i \geq 1$	Fragmentation	computed as $D_i/D_i^\star$.
$\phi_i \geq 1$	Polycentrism	weighted by the volume of each branch in the contour density of the city
$\theta_i \geq 60,000$	Footprint	given by $B_i A_i$ in m^2
$\Delta_i \geq 0$	Pyramid	ratio between the average size of buildings within 1 km of the densest point of a city and the average size in that city A_i.
$T_i^{(R)} \geq 1$	Emptiness	sprawl inside a circle of radius R centred at the most dense location of the city.

Table 14: Terms summary

Observed results at a regional level in North, West, East, Central and South Africa (Table 15).

References

[1] François Moriconi-Ebrard, Dominique Harre, and Philipp Heinrigs. Urbanisation dynamics in West Africa 1950 - 2010, 2015.

[2] Wojciech Sirko, Sergii Kashubin, Marvin Ritter, Abigail Annkah, Yasser Salah Eddine Bouchareb, Yann Dauphin, Daniel Keysers, Maxim Neumann, Moustapha Cisse, and John Quinn. Continental-scale building detection from high resolution satellite imagery. *arXiv preprint arXiv:2107.12283*, 1, 2021.

[3] R Core Team. R: A language and environment for statistical computing, 2018.

[4] Thomas Esch, Elisabeth Brzoska, Stefan Dech, Benjamin Leutner, Daniela Palacios-Lopez, Annekatrin Metz-Marconcini, Mattia Marconcini, Achim Roth, and Julian Zeidler. World Settlement Footprint 3D-a first three-dimensional survey of the global building stock. *Remote Sensing of Environment*, 270:112877, 2022.

[5] Geoff Boeing. OSMnx: New methods for acquiring, constructing, analyzing, and visualizing complex street networks. *Computers, Environment and Urban Systems*, 65:126–139, 2017.

[6] NASA JPL. NASADEM Merged DEM Global 1 arc second V001 [Data set]., 2020.

[7] Michelle Parry and Ephraim Fischbach. Probability distribution of distance in a uniform ellipsoid: Theory and applications to physics. *Journal of Mathematical Physics*, 41(4):2417–2433, 2000.

[8] Luis MA Bettencourt, Jose Lobo, Dirk Helbing, Christian Kuhnert, and Geoffrey B West. Growth, innovation, scaling, and the pace of life in cities. *PNAS*, 104(17):7301–7306, 2007.

[9] Rafael Prieto-Curiel, Luca Pappalardo, Lorenzo Gabrielli, and Steven Richard Bishop. Gravity and scaling laws of city to city migration. *PloS one*, 13(7):e0199892, 2018.

[10] Daniel Godoy-Shimizu, Philip Steadman, Ian Hamilton, Michael Donn, Stephen Evans, Graciela Moreno, and Homeira Shayesteh. Energy use and height in office buildings. *Building Research & Information*, 46(8):845–863, 2018.

[11] Ian Hamilton, Stephen Evans, Philip Steadman, Daniel Godoy-Shimizu, Michael Donn, Homeira Shayesteh, and Graciela Moreno. All the way to the top! The energy implications of building tall cities. *Energy Procedia*, 122:493–498, 2017.

[12] Toni Tukia, Semen Uimonen, Marja-Liisa Siikonen, Claudio Donghi, and Matti Lehtonen. Modeling the aggregated power consumption of elevators–the New York City case study. *Applied Energy*, 251:113356, 2019.

[13] Daniel Godoy-Shimizu, Philip Steadman, and Stephen Evans. Density and morphology: from the building scale to the city scale. *Buildings & Cities*, 2(1):92–113, 2021.

[14] Shlomo Angel, Patrick Lamson-Hall, and Zeltia Gonzalez Blanco. Anatomy of density: measurable factors that constitute urban density. *Buildings and Cities*, 2(1), 2021.

[15] Karen C Seto, Shobhakar Dhakal, Anthony Bigio, Hilda Blanco, Gian Carlo Delgado, David Dewar, Luxin Huang, Atsushi Inaba, Arun Kansal, Shuaib Lwasa, et al. Human settlements, infrastructure and spatial planning. *Climate Change 2014: Mitigation of Climate Change, Intergovernmental Panel on Climate Change IPCC*, 1, 2014.

[16] Ramana Gudipudi, Diego Rybski, Matthias KB Lüdeke, Bin Zhou, Zhu Liu, and Jürgen P Kropp. The efficient, the intensive, and the productive: Insights from urban Kaya scaling. *Applied Energy*, 236:155–162, 2019.

[17] Shlomo Angel and Patrick Lamson-Hall. Anatomy of density II: A comprehensive strategy for making room for city densification. *SSRN 3744466*, 2020.

[18] Daniel Hoornweg and Kevin Pope. Population predictions for the worlds largest cities in the 21st century. *Environment and Urbanization*, 29(1):195–216, 2017.

[19] Denise Pumain and Marianne Guerois. Scaling laws in urban systems. In *Santa Fe Institute, Working Papers*, pages 1–26, 2004.

[20] Jules Depersin and Marc Barthelemy. From global scaling to the dynamics of individual cities. *Proceedings of the National Academy of Sciences*, 115(10):2317–2322, 2018.

[21] Rafael Prieto-Curiel, Abel Schumann, Inhoi Heo, and Philipp Heinrigs. Detecting cities with high intermediacy in the African urban network. *Computers, Environment and Urban Systems*, 98:101869, 2022.

[22] Rafael Prieto-Curiel, Humberto González Ramírez, Mauricio Quiñones Domínguez, and Juan Pablo Orjuela Mendoza. A paradox of traffic and extra cars in a city as a collective behaviour. *Royal Society Open Science*, 8(6):201808, 2021.

	$y = logB_N$	$y = logB_W$	$y = logB_E$	$y = logB_C$	$y = logB_S$
(Intercept)	-0.370^{**}	-0.715^{***}	-1.388^{***}	-1.038^{***}	-0.472^{**}
	(0.135)	(0.116)	(0.107)	(0.181)	(0.148)
log(Population_2015)	0.926^{***}	0.957^{***}	1.042^{***}	0.990^{***}	1.005^{***}
	(0.013)	(0.011)	(0.010)	(0.017)	(0.014)
R^2	0.763	0.792	0.886	0.889	0.916
	$y = logA_N$	$y = logA_W$	$y = logA_E$	$y = logA_C$	$y = logA_S$
(Intercept)	-10.300^{***}	-11.264^{***}	-10.413^{***}	-10.992^{***}	-10.151^{***}
	(0.095)	(0.102)	(0.072)	(0.131)	(0.109)
log(Population_2015)	0.044^{***}	0.144^{***}	0.035^{***}	0.076^{***}	0.018
	(0.009)	(0.010)	(0.007)	(0.012)	(0.010)
R^2	0.015	0.100	0.019	0.082	0.007
	$y = logS_N$	$y = logS_W$	$y = logS_E$	$y = logS_C$	$y = logS_S$
(Intercept)	-1.366^{***}	-0.073	-0.351^{*}	1.528^{***}	0.843^{***}
	(0.128)	(0.095)	(0.136)	(0.147)	(0.192)
log(Population_2015)	0.106^{***}	-0.026^{**}	0.026^{*}	-0.156^{***}	-0.078^{***}
	(0.012)	(0.009)	(0.013)	(0.014)	(0.019)
R^2	0.045	0.004	0.003	0.231	0.038
	$y = logE_N$	$y = logE_W$	$y = logE_E$	$y = logE_C$	$y = logE_S$
(Intercept)	0.964^{***}	1.909^{***}	2.094^{***}	3.150^{***}	2.514^{***}
	(0.100)	(0.074)	(0.108)	(0.153)	(0.200)
log(Population_2015)	0.071^{***}	-0.017^{*}	-0.001	-0.109^{***}	-0.065^{***}
	(0.010)	(0.007)	(0.010)	(0.015)	(0.019)
R^2	0.033	0.003	0.000	0.120	0.025
	$y = log\psi_N$	$y = log\psi_W$	$y = log\psi_E$	$y = log\psi_C$	$y = log\psi_S$
(Intercept)	-0.201	0.918^{***}	0.872^{***}	2.339^{***}	1.679^{***}
	(0.108)	(0.078)	(0.113)	(0.132)	(0.189)
log(Population_2015)	0.089^{***}	-0.022^{**}	0.013	-0.133^{***}	-0.072^{***}
	(0.010)	(0.008)	(0.011)	(0.013)	(0.018)
R^2	0.044	0.004	0.001	0.213	0.033
	$y = log\phi_N$	$y = log\phi_W$	$y = log\phi_E$	$y = log\phi_C$	$y = log\phi_S$
(Intercept)	-2.959^{***}	-1.791^{***}	-3.168^{***}	-0.999^{***}	-2.435^{***}
	(0.090)	(0.058)	(0.102)	(0.080)	(0.142)
log(Population_2015)	0.310^{***}	0.186^{***}	0.325^{***}	0.103^{***}	0.259^{***}
	(0.009)	(0.006)	(0.010)	(0.008)	(0.014)
R^2	0.448	0.363	0.458	0.311	0.441
	$y = logD_N$	$y = logD_W$	$y = logD_E$	$y = logD_C$	$y = logD_S$
(Intercept)	-5.635^{***}	-5.170^{***}	-5.128^{***}	-3.776^{***}	-3.732^{***}
	(0.128)	(0.085)	(0.111)	(0.130)	(0.226)
log(Population_2015)	0.574^{***}	0.528^{***}	0.551^{***}	0.400^{***}	0.440^{***}
	(0.012)	(0.008)	(0.011)	(0.012)	(0.022)
R^2	0.579	0.681	0.672	0.717	0.474
	$y = logD^{\star}_N$	$y = logD^{\star}_W$	$y = logD^{\star}_E$	$y = logD^{\star}_C$	$y = logD^{\star}_S$
(Intercept)	-5.434^{***}	-6.088^{***}	-6.000^{***}	-6.114^{***}	-5.411^{***}
	(0.072)	(0.048)	(0.057)	(0.097)	(0.094)
log(Population_2015)	0.485^{***}	0.550^{***}	0.539^{***}	0.533^{***}	0.512^{***}
	(0.007)	(0.005)	(0.006)	(0.009)	(0.009)
R^2	0.760	0.879	0.880	0.891	0.877
	$y = logA100_N$	$y = logA100_W$	$y = logA100_E$	$y = logA100_C$	$y = logA100_S$
(Intercept)	-1.590^{***}	-2.886^{***}	-2.477^{***}	-3.744^{***}	-1.412^{***}
	(0.148)	(0.134)	(0.138)	(0.254)	(0.178)
log(Population_2015)	0.078^{***}	0.201^{***}	0.122^{***}	0.204^{***}	0.048^{**}
	(0.014)	(0.013)	(0.013)	(0.024)	(0.017)
R^2	0.019	0.111	0.061	0.148	0.017
	$y = logFP1km_N$	$y = logFP1km_W$	$y = logFP1km_E$	$y = logFP1km_C$	$y = logFP1km_S$
(Intercept)	8.233^{***}	7.619^{***}	8.033^{***}	6.767^{***}	8.250^{***}
	(0.138)	(0.100)	(0.130)	(0.201)	(0.223)
log(Population_2015)	0.408^{***}	0.471^{***}	0.413^{***}	0.515^{***}	0.402^{***}
	(0.013)	(0.010)	(0.012)	(0.019)	(0.022)
R^2	0.375	0.551	0.454	0.638	0.435
	$y = logFP_N$	$y = logFP_W$	$y = logFP_E$	$y = logFP_C$	$y = logFP_S$
(Intercept)	3.146^{***}	1.837^{***}	2.015^{***}	1.786^{***}	3.193^{***}
	(0.143)	(0.096)	(0.114)	(0.193)	(0.187)
log(Population_2015)	0.970^{***}	1.100^{***}	1.078^{***}	1.066^{***}	1.023^{***}
	(0.014)	(0.009)	(0.011)	(0.018)	(0.018)
R^2	0.760	0.879	0.880	0.891	0.877
	$y = logSOpenGreen_N$	$y = logSOpenGreen_W$	$y = logSOpenGreen_E$	$y = logSOpenGreen_C$	$y = logSOpenGreen_S$
(Intercept)	-0.543	0.010	-0.015	2.414^{***}	1.894^{***}
	(0.489)	(0.238)	(0.252)	(0.301)	(0.482)
log(Population_2015)	1.018^{***}	1.142^{***}	1.163^{***}	0.926^{***}	1.030^{***}
	(0.047)	(0.023)	(0.024)	(0.029)	(0.047)
R^2	0.230	0.561	0.638	0.717	0.521
Num. obs.	1547	1898	1312	416	452

$^{***}p < 0.001$; $^{**}p < 0.01$; $^{*}p < 0.05$

Table 15: Coefficients across regions in Africa
